# Cannabidiol selectively downregulates FAM111B expression and induces apoptosis in fibrosarcoma cells: an in vitro study

**DOI:** 10.1186/s42238-026-00481-1

**Published:** 2026-08-01

**Authors:** Danielle Naicker, Falone Sunda, Cenza Rhoda, Hygon Mutavhatsindi, Nonhlanhla Khumalo, Afolake Arowolo

**Affiliations:** 1 Hair and Skin Research Laboratory, Division of Dermatology, Department of Medicine, University of Cape Town, Cape Town, South Africa; 2 Biomedical Research and Innovation Platform, South African Medical Research Council, Tygerberg, South Africa; 3 Department of Medicine, Faculty of Health Sciences, University of Cape Town, Cape Town, South Africa

**Keywords:** Cannabidiol, Fibrosarcoma, FAM111B, BAG3, Apoptosis, Cell-cycle, Flow cytometry

## Abstract

**Background:**

Fibrosarcoma is a rare and aggressive soft tissue sarcoma with limited therapeutic options and poor outcomes in advanced disease. Family with sequence similarity 111 member B (FAM111B) has emerged as a pro-tumorigenic factor associated with dysregulated cell survival and proliferation. Cannabidiol (CBD), a non-psychoactive phytocannabinoid, exhibits anticancer activity in multiple malignancies, but its effects on FAM111B expression and downstream pathways in fibrosarcoma remain unclear.

**Methods:**

The effects of CBD were evaluated in human fibrosarcoma (HT1080) cells and non-cancerous human fibroblasts (FG0). Cell metabolic activity was assessed using an MTT assay. Gene and protein expression were determined by real-time quantitative PCR and Western blotting, respectively. Apoptosis and cell-cycle distribution were assessed by flow cytometry using Annexin V-FITC/propidium iodide (PI) and PI staining alone, respectively. Statistical analyses were performed using an unpaired Student’s t-test or a two-way analysis of variance (ANOVA) followed by Šídák’s post hoc multiple comparisons test, as appropriate.

**Results:**

CBD reduced metabolic activity in both HT1080 and FG0 cells in a dose-dependent manner, with comparable IC₅₀ values (28.95 ± 3.15 µM and 30.75 ± 9.12 µM, respectively), indicating limited selectivity based on metabolic activity alone. CBD significantly reduced *FAM111B* mRNA expression by approximately 3-fold in HT1080 cells and 2-fold in FG0 cells; however, a significant reduction in FAM111B protein expression was observed only in HT1080 cells, CBD treatment was associated with marked downregulation of BAG3 and Cyclin B1 proteins, increased apoptosis (Annexin V-positive cells: 3.55 ± 0.78% vs. 13.20 ± 4.56%), and altered cell-cycle progression characterized by increased Sub-G1 (3.44 ± 1.10% vs. 18.37 ± 0.83%) and G0/G1 (28.30 ± 1.57% vs. 39.17 ± 0.85%) populations, with corresponding reductions in the S- and G2/M-phase populations. These effects were markedly attenuated in non-cancerous fibroblasts.

**Conclusions:**

CBD exerts context-dependent anticancer effects in HT1080 fibrosarcoma cells by selectively suppressing FAM111B protein expression, promoting apoptosis, and disrupting cell-cycle progression. These findings identify FAM111B- and BAG3-associated pathways as potential therapeutic vulnerabilities and support further investigation of CBD as a modulator of stress-adaptive and proliferative signaling in preclinical fibrosarcoma models.

**Supplementary Information:**

The online version contains supplementary material available at https://doi.org/10.1186/s42238-026-00481-1.

## Introduction

Fibrosarcoma, also known as fibroblastic sarcoma, is a malignant mesenchymal tumor characterized by the proliferation of immature spindle-shaped fibroblasts (Ferreira et al. 2013, Sultana and Sailaja 2025, Augsburger et al. 2017). While historically considered a common soft tissue sarcoma, reclassification of tumor subtypes has refined its diagnosis, now accounting for less than 1% of all adult malignancies (Sultana and Sailaja 2025, Bansal et al. 2021). Despite advances in surgical resection and adjuvant therapies, the prognosis for advanced or metastatic fibrosarcoma remains poor owing to frequent local recurrence and resistance to conventional chemotherapy (Sahin et al. 2026, Gómez-Puerto et al. 2025). Consequently, there is an urgent need to identify novel molecular targets and therapeutic agents that can disrupt tumorigenesis while minimizing systemic toxicity.

Recent genomic and functional studies have implicated the *Family with sequence similarity 111 member B (FAM111B)* gene as a pro-tumorigenic factor across multiple cancer types (Sun et al. 2019, Rhoda et al. 2023, Wu and Liang 2023, Wang et al. 2025, Tomioka et al. 2024). Mechanistically, dysregulated FAM111B activity has been linked to the promotion of DNA replication and repair under stress conditions (Hoffmann et al. 2020, Wei et al. 2025), maintenance of genome stability (Roversi et al. 2021), and the modulation of pro-survival signaling pathways, including PI3K/Akt (Wang et al. 2025, Yang et al. 2024, Wang et al. 2023), thereby contributing to tumor growth and resistance to apoptosis in certain malignancies. Beyond its role in genome maintenance, knockout or dysregulation of FAM111B alters the expression of genes involved in p53-associated signaling, including *BAG3*, *BCL2*, *CDKN1A*, and *CCNB1*, thereby implicating FAM111B in the regulation of apoptosis and cell cycle-progression [as synthesized in (Arowolo et al. 2022, Naicker et al. 2024) ]. Although FAM111B overexpression has been reported in HT1080 fibrosarcoma cells (Rhoda et al. 2023), its functional role in fibrosarcoma tumorigenesis and its therapeutic potential remain underexplored.

Cannabidiol (CBD) is a non-psychoactive phytocannabinoid derived from Cannabis sativa with well-documented antifibrosis and anticancer properties (Sunda and Arowolo 2020, Ma et al. 2024). Notably, accumulating evidence indicates that CBD exerts anti-tumor effects by inhibiting the PI3K/Akt/mTOR signaling axis (Tong et al. 2025), a pathway previously implicated in FAM111B-mediated tumor growth (Wang et al. 2025, Yang et al. 2024, Wang et al. 2023). Despite this mechanistic overlap, no study to date has investigated whether CBD modulates FAM111B expression in fibrosarcoma.

Accordingly, this study investigated the effects of CBD on FAM111B expression and downstream apoptosis- and cell-cycle-associated pathways in a human fibrosarcoma cell model, with the hypothesis that CBD suppresses FAM111B expression, resulting in molecular and phenotypic changes associated with impaired cell survival and proliferation. To test this hypothesis, the effects of CBD on FAM111B expression, apoptosis, and cell-cycle-associated markers (BAG3, Bcl-2, p21, Cyclin B1, and p53) were evaluated, along with the resulting cellular responses.

## Methods

### Cell lines

Human fibrosarcoma (HT1080; ATCC CCL-121) cells were obtained from the American Type Culture Collection (ATCC). The FG0 cell line consists of primary, non-cancerous human dermal fibroblasts originally obtained from the Department of Medicine at the University of Cape Town, South Africa, and kindly provided by the Denver Laboratory. FG0 cells were used as the non-cancerous fibroblast control throughout the study. Both cell lines were maintained in Dulbecco’s Modified Eagle Medium (DMEM; Gibco™, Thermo Fisher Scientific, Waltham, MA, USA) supplemented with 10% (v/v) fetal bovine serum (FBS; Gibco™, Thermo Fisher Scientific, Waltham, MA, USA) and 1% penicillin–streptomycin (Gibco™, Thermo Fisher Scientific, Waltham, MA, USA). Cells were incubated at 37 °C in a humidified incubator with 5% CO₂ and were routinely passaged at 70–80% confluence.

### Chemical compounds

Cannabidiol (CBD; analytical standard, ≥ 98.5% purity) was purchased from Sigma-Aldrich (St. Louis, MO, USA; catalog no. 90899) as a solution in ethanol (10 mg/mL). Aliquots were stored at − 80 °C and diluted in complete culture medium immediately before use. Vehicle control cells received an equivalent concentration of ethanol (Sigma-Aldrich, St. Louis, MO, USA) corresponding to the highest ethanol concentration used in CBD-treated samples. The final ethanol concentration did not exceed 0.1% (v/v) and was kept constant across all experimental conditions.

### MTT assay

HT1080 and FG0 cells were seeded into 96-well plates at a density of 10 000 cells per well and allowed to adhere for 24 h. Cells were then treated with increasing concentrations of CBD (0-500 µM; serial dilution) for another 24 h. Vehicle control wells received an equivalent concentration of ethanol (≤ 0.1% v/v).

Cell metabolic activity was assessed using the 3-(4,5-dimethylthiazol-2-yl)-2,5-diphenyltetrazolium Bromide (MTT) Assay Kit (Sigma-Aldrich, St. Louis, MO, USA; Cat. No. CT01) according to the manufacturer’s protocol. Briefly, 10 µL of MTT reagent was added to each well, followed by incubation at 37 °C for 4 h to allow formazan crystals to form. Subsequently, 100 µL of isopropanol containing 0.04 N HCl was added to each well to solubilize the formazan crystals, and plates were mixed thoroughly using a plate mixer.

Absorbance was measured within 1 h using a microplate reader at 570 nm with a reference wavelength of 630 nm. Cell metabolic activity was normalized to the corresponding untreated vehicle control and expressed as a percentage of control. The half-maximal inhibitory concentration (IC₅₀) values were estimated by nonlinear regression analysis using GraphPad Prism 10 (GraphPad Software, San Diego, CA, USA). The selectivity index was calculated as the ratio of the IC₅₀ values for FG0 and HT1080 cells.

Each experiment included eight technical replicates per condition to minimize intra-assay variability and improve the precision of absorbance measurements. Experiments were independently repeated at least three times using different cell culture passages to generate biological replicates and assess reproducibility.

### Quantitative real-time polymerase chain reaction (qPCR)

Total RNA was extracted from CBD-treated and untreated HT1080 and FG0 cells using the GeneJET RNA Purification Kit (Thermo Scientific™, Vilnius, Lithuania) according to the manufacturer’s instructions. RNA concentration and purity were assessed spectrophotometrically, and only samples with acceptable purity were used for downstream analysis. Complementary DNA (cDNA) was synthesized from 1 µg of total RNA using the LunaScript RT SuperMix Kit (New England Biolabs, Ipswich, MA, USA).

Quantitative real-time PCR was performed using Luna Universal qPCR Master Mix (New England Biolabs) on a QuantStudio™ 6 Flex Real-Time PCR System (Thermo Fisher Scientific, Waltham, MA, USA) according to the manufacturer’s recommended cycling conditions. Gene-specific primers were used as listed in Table 1. Primers for FAM111B were designed in-house, verified using NCBI BLAST to confirm target specificity, and synthesized by Inqaba Biotec (Pretoria, South Africa). All reactions were performed in technical triplicate. Amplification specificity was confirmed by melt curve analysis.

**Table 1:** Primer sequences used for quantitative real-time PCR (qPCR)

	Forward primer	Reverse primer	Accession
FAM111B	GACCGTAGTGTGTTTACAGCA	GAAATAGTCATTAGCACTCAGGGC	NM_001142704.2
*BAG3*	CTCCATTCCGGTGATACACGA	TGGTGGGTCTGGTACTCCC	NM_004281.4
*BCL2* *(Bcl-2)*	GATGTGATGCCTCTGCGAAG	CATGCTGATGTCTCTGGAAT CT	NM_000633.3
*CCNB1* *(Cyclin B1)*	CAGTCAGACCAAAATACCTAC TGGGT	ACACCAACCAGCTGCAGCATCT TCTT	NM_031966.4
*CDKN1A* (*p21)*	AAGACCATGTGGACCTGT	GGTAGAAATCTGTCATGCTG	NM_001374511.1
TP53 (*p53)*	TGCGTGTGGAGTATTTGGATG	TGGTACAGTCAGAGCCAACCTC	NM_000546.6
*GAPDH*	GTCTCCTCTGACTTCAACAGCG	ACCACCCTGTTGCTGTAGCCAA	NM_002046
*ACTB*	TGTCACCAACTGGGACGATA	TCTCAGCTGTGGTGGTGAAG	NM_001017992.4

Gene expression levels were normalized to *GAPDH* and *ACTB*, which have been validated as stable internal reference genes in similar cancer cell models (Kose 2024), and relative expression was calculated using the 2⁻ΔΔCq method (Livak and Schmittgen 2001).

### Western blot analysis

Total protein was extracted from CBD-treated (24-hour treatment at the respective IC_50_ concentrations) and untreated HT1080 and FG0 cells using RIPA Extraction Buffer (Thermo Fisher Scientific, Waltham, MA, USA) supplemented with cOmplete™ Protease Inhibitor Cocktail (Roche Diagnostics, Basel, Switzerland). Protein concentration was determined using the bicinchoninic acid (BCA) assay (Pierce™, Thermo Fisher Scientific, Waltham, MA, USA). Equal amounts of protein (~ 20 µg) were resolved by electrophoresis on 10% SDS polyacrylamide gels and transferred onto polyvinylidene difluoride (PVDF) membranes.

Membranes were blocked in 5% non-fat dry milk in Tris-buffered saline containing 0.1% Tween-20 and incubated with primary antibodies against FAM111B (rabbit polyclonal antibody, HPA038637; 1:5000, Sigma-Aldrich, St. Louis, MO, USA), BAG3 (goat polyclonal antibody, PA5-18061; 1:1000, Invitrogen™, Thermo Fisher Scientific, Waltham, MA, USA), Cyclin B1 (GNS1I mouse monoclonal antibody, MA5-14327; 1:1000, Invitrogen™, Thermo Fisher Scientific, Waltham, MA, USA), p53 (7F5 rabbit monoclonal antibody, #2527; 1:1000, Cell Signaling Technology, Danvers, MA, USA), Bcl-2 (OTI9D3 mouse mAb, TA806591; 1:2000, Invitrogen™, Thermo Fisher Scientific, Waltham, MA, USA), p21 (12D1 rabbit mAb, #2947; 1:1000, Cell Signaling Technology, Danvers, MA, USA), and GAPDH (14C10 rabbit mAb, #2118; 1:1000, Cell Signaling Technology, Danvers, MA, USA) overnight at 4 °C. PVDF membranes were then incubated with the appropriate HRP-conjugated secondary antibodies for 1 h at room temperature.

Protein bands were visualized using enhanced chemiluminescence (Clarity™ Western ECL Substrate, Bio-Rad Laboratories, Hercules, CA, USA) and imaged using the ChemiDoc™ Imaging System (Bio-Rad Laboratories, Hercules, CA, USA). Protein expression was quantified by densitometric analysis using ImageJ software (National Institutes of Health, Bethesda, MD, USA) following normalization to the corresponding GAPDH loading control. For each independent biological experiment, protein lysates from untreated and CBD-treated cells were analyzed in triplicate (technical replicates). Representative Western blot images from one of three independent biological experiments are shown, and quantitative data are presented as the mean ± SD of three independent biological experiments.

### Flow cytometry

#### Apoptosis analysis

Apoptosis was assessed using the Annexin V-FITC Apoptosis Detection Kit (Roche Diagnostics, Basel, Switzerland) in combination with propidium iodide (PI; Thermo Fisher Scientific, Waltham, MA, USA) according to the manufacturer’s instructions. Following 24 h of CBD treatment, HT1080 and FG0 cells were harvested, washed twice with phosphate-buffered saline (PBS), and resuspended in 1× binding buffer (0.1 M HEPES, pH 7.4; 1.4 M NaCl; 25 mM CaCl₂). Cells were stained with Annexin V-FITC and propidium iodide (PI) and incubated for 15 min at room temperature protected from light according to the manufacturers’ instructions.

Appropriate controls, including unstained cells, Annexin V-FITC-only stained cells, and PI-only stained cells, were used to establish compensation settings and define gating boundaries. Samples were analyzed using a BD LSRFortessa flow cytometer (BD Biosciences, San Jose, CA, USA), with approximately 10,000 events collected per sample. Flow cytometry data were analyzed using FlowJo software (version 10; BD Biosciences).

Cell populations were classified as viable (Annexin V⁻/PI⁻), early apoptotic (Annexin V⁺/PI⁻), late apoptotic (Annexin V⁺/PI⁺), or necrotic (Annexin V⁻/PI⁺). For quantitative analyses, total apoptotic cells were calculated as the sum of the early and late apoptotic populations and expressed as the percentage of total acquired events.

#### Cell cycle analysis

HT1080 and FG0 cells were treated with CBD for 24 h or left untreated, harvested by centrifugation and stained with PI/RNase Staining Buffer (Roche Diagnostics, Basel, Switzerland) according to the manufacturer’s instructions for cell-cycle analysis. Cells were incubated with the staining solution for 30 min at room temperature in the dark, then analyzed using a BD LSRFortessa flow cytometer (BD Biosciences, San Jose, CA, USA).

Single-cell events were identified by gating on forward-scatter area versus forward-scatter height prior to DNA-content analysis. Following exclusion of debris and cell aggregates, DNA-content histograms were analyzed using FlowJo software (version 10; BD Biosciences). Cell-cycle phase distributions were determined using the Dean–Jett–Fox modeling algorithm, which was used for the primary quantitative analysis presented in the main manuscript. The model-derived “% less G1” population was designated as the Sub-G1 fraction, while the G2/M fraction was calculated as the sum of the model-derived G2 and “% greater G2” populations. Cell-cycle phase distributions were also analyzed independently using manual gating as a complementary approach. The proportions of cells in the Sub-G1, G0/G1, S, and G2/M phases were compared between untreated and CBD-treated cells.

### Data and statistical analysis

Statistical analyses were performed using GraphPad Prism 10 (GraphPad Software, San Diego, CA, USA). Microsoft Excel 2019 (Microsoft Corporation, Redmond, WA, USA) was used to organize data. Comparisons between CBD-treated and corresponding control groups were conducted using an unpaired two-tailed Student’s t-test. Cell-cycle phase distributions were analyzed separately for each cell line using two-way analysis of variance (ANOVA), followed by Šídák’s multiple-comparisons test. Post hoc pairwise comparisons were performed between untreated and CBD-treated groups within each cell-cycle phase. A value of *p* < 0.05 was considered statistically significant, with significance levels indicated as *p* < 0.05 (*), *p* < 0.01 (**), and *p* < 0.001 or *p* < 0.0001 (***).

## Results

### CBD does not exhibit strong selectivity between fibrosarcoma and non-cancerous fibroblast cell lines

**Fig. 1:**
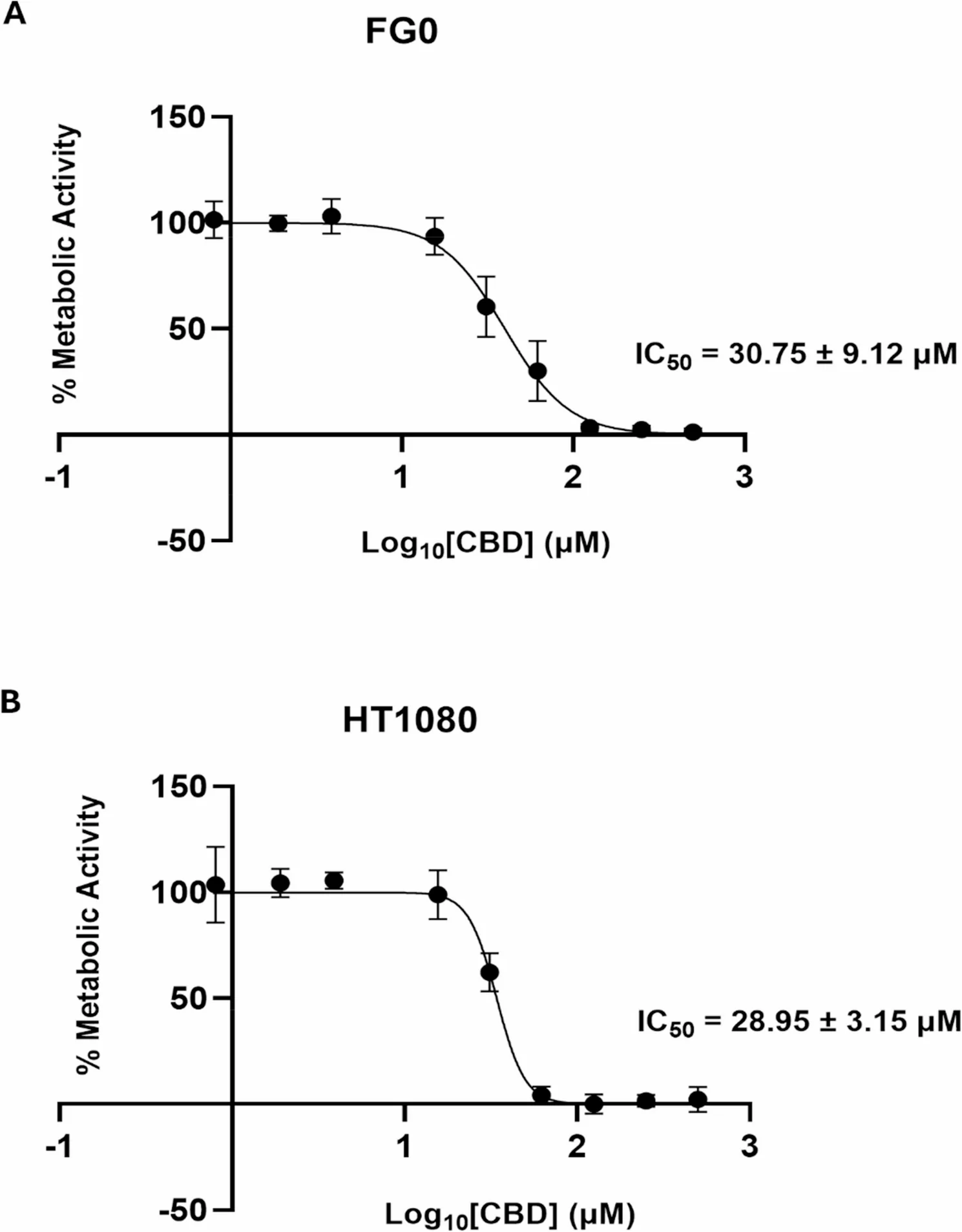
CBD reduces cell metabolic activity in both FG0 and HT1080 cells with comparable potency. Dose–response curves showing the effects of CBD on cell metabolic activity in FG0 (**A**) and HT1080 (**B**) cells following 24 h treatment, as determined by the MTT assay. Cell metabolic activity is expressed relative to untreated controls. Dose–response curves are plotted against the logarithm of CBD concentration. IC₅₀ values were calculated from nonlinear regression analyses and used to determine the concentrations selected for subsequent experiments. Data are presented as mean ± SD from three independent biological experiments

CBD treatment produced a dose-dependent reduction in cell metabolic activity in both FG0 fibroblasts and HT1080 fibrosarcoma cells following 24 h of exposure, as determined by the MTT assay (Fig. 1A and B). The calculated IC₅₀ values across three independent biological experiments were 30.75 ± 9.12 µM for FG0 cells and 28.95 ± 3.15 µM for HT1080 cells. Based on these values, CBD concentrations of 30.75 µM (FG0) and 28.95 µM (HT1080) were selected for subsequent analyses. The calculated selectivity index was 1.06, indicating limited selectivity between the two cell lines based on cell metabolic activity. Representative bright-field images of untreated and CBD-treated FG0 and HT1080 cells are provided in Supplementary Figure S1.

### CBD reduces FAM111B mRNA expression in both cell lines, but selectively decreases FAM111B protein levels in HT1080 cells

**Fig. 2:**
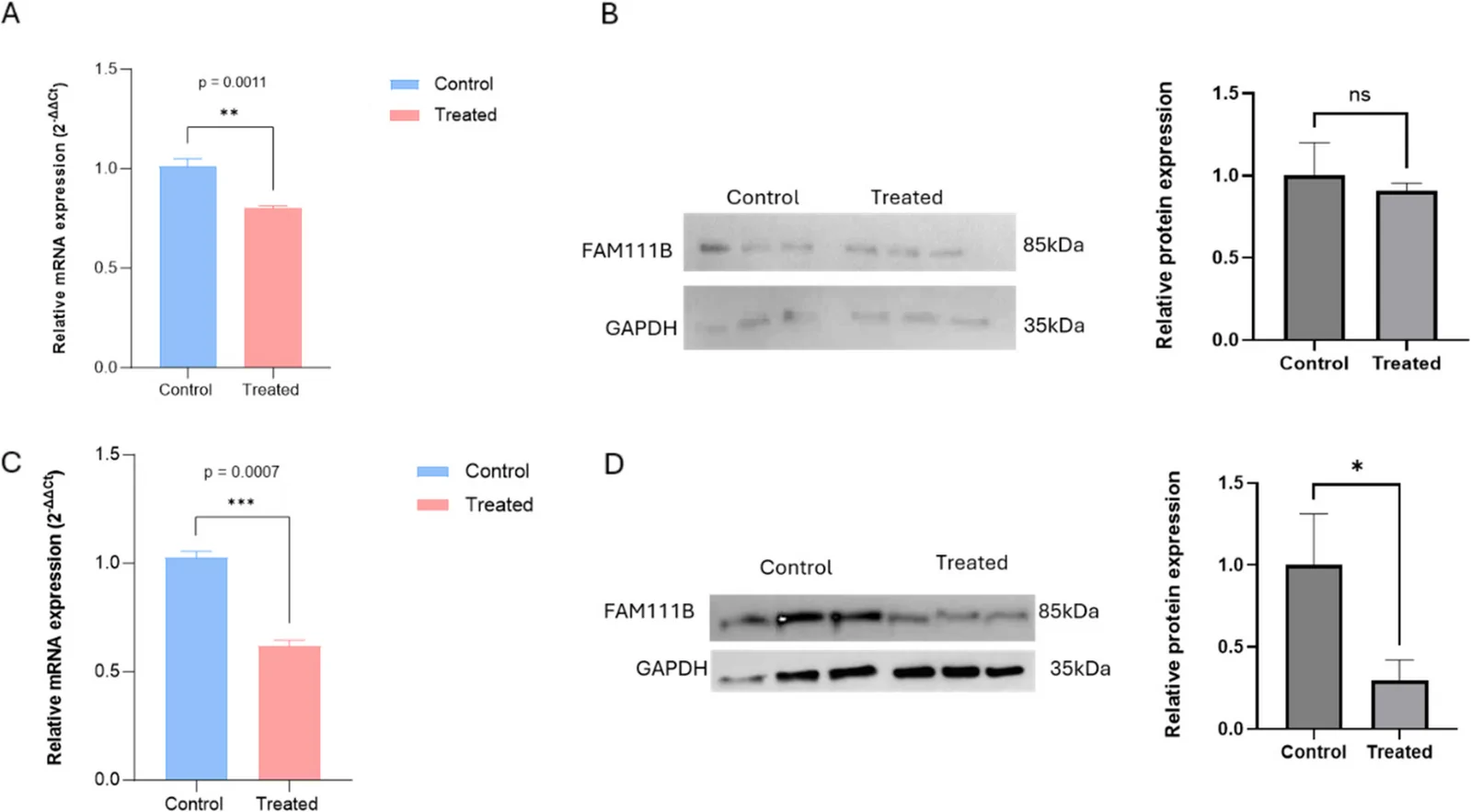
CBD reduced FAM111B mRNA expression in both non-cancerous fibroblast (FG0) and fibrosarcoma (HT1080) cells, but selectively decreased FAM111B protein expression in HT1080 cells. **A** qPCR analysis of FAM111B mRNA expression in FG0 cells following CBD treatment. **B** Representative Western blot image and corresponding densitometric analysis of FAM111B protein expression in FG0 cells. **C** qPCR analysis of FAM111B mRNA expression in HT1080 cells following CBD treatment. **D** Representative Western blot image and corresponding densitometric analysis of FAM111B protein expression in HT1080 cells. Representative Western blot images are shown from one of three independent biological experiments, with protein samples loaded in triplicate (technical replicates). Densitometric data are presented as mean ± SD from three independent biological experiments following normalization to GAPDH. Statistical significance is indicated as *p* < 0.05 (*), *p* < 0.01 (**), and *p* < 0.001 or *p* < 0.0001 (***)

CBD-mediated changes in FAM111B expression were assessed at both the mRNA and protein levels. Quantitative PCR analysis demonstrated a significant reduction in *FAM111B* mRNA expression following CBD treatment in both FG0 and HT1080 cells, with decreases of approximately 2-fold and 3-fold, respectively (Fig. 2A and C).

At the protein level, however, the response differed between the two cell lines. Despite the reduction in *FAM111B* mRNA expression, CBD treatment did not significantly alter FAM111B protein abundance in FG0 cells relative to untreated controls (Fig. 2B). In contrast, HT1080 fibrosarcoma cells exhibited a significant reduction in FAM111B protein levels following CBD treatment (Fig. 2D).

Together, these results indicate that although CBD suppresses *FAM111B* mRNA expression in both FG0 and HT1080 cells, a corresponding reduction in FAM111B protein abundance occurs selectively in HT1080 fibrosarcoma cells, suggesting differential post-transcriptional and/or post-translational regulation between the two cell types.

### CBD differentially alters apoptosis- and cell cycle-associated markers in HT1080 but not FG0 cells

**Fig. 3:**
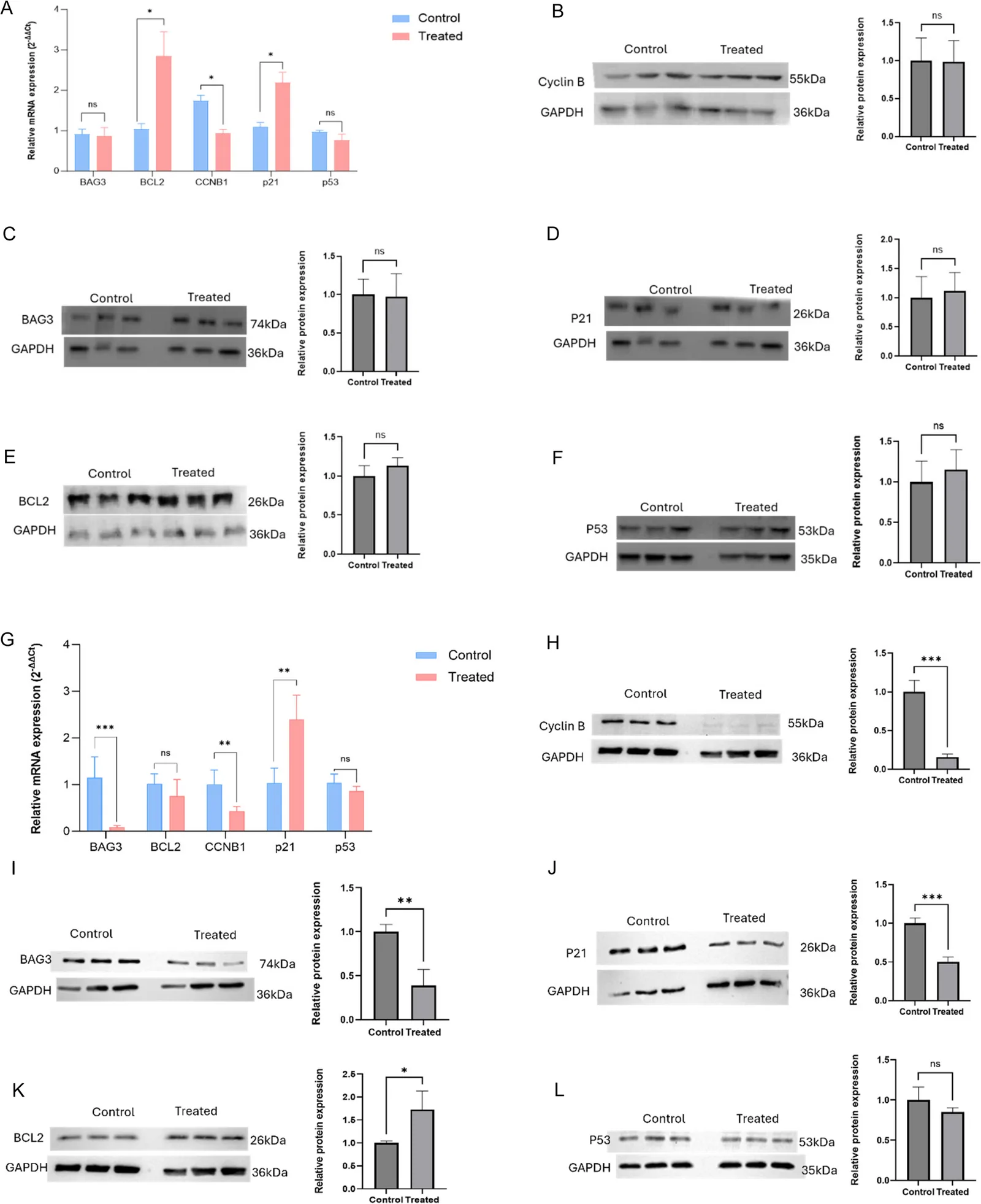
CBD differentially regulated apoptosis- and cell cycle-associated markers in non-cancerous fibroblast (FG0) and fibrosarcoma (HT1080) cells. **A** qPCR analysis of BAG3, BCL2, CCNB, CDKN1A (p21), and TP53 (p53) mRNA expression in FG0 cells following CBD treatment. **B**–**F** Representative Western blot images and corresponding densitometric analyses of BAG3, Bcl-2, Cyclin B1, p21, and p53 protein expression in FG0 cells. **G** qPCR analysis of BAG3, BCL2, CCNB1, CDKN1A (p21), and TP53 (p53) mRNA expression in HT1080 cells following CBD treatment. **H**–**L** Representative Western blot images and corresponding densitometric analyses of BAG3, Bcl-2, Cyclin B1, p21, and p53 protein expression in HT1080 cells. Representative Western blot images are shown from one of three independent biological experiments, with protein samples loaded in triplicate (technical replicates). Densitometric data are presented as mean ± SD from three independent biological experiments following normalization to GAPDH. Statistical significance is indicated as *p* < 0.05 (*), *p* < 0.01 (**), and *p* < 0.001 or *p* < 0.0001 (***)

CBD-induced changes in the expression of apoptosis- and cell cycle-related markers were evaluated by qPCR and Western blot analysis. In FG0 cells, CBD treatment resulted in a significant reduction in *CCNB1* mRNA expression (approximately 2-fold, *p* < 0.05) and a significant increase in *CDKN1A (p21)* mRNA expression (Fig. 3A). No significant changes were observed in *BAG3* or *TP53* transcript levels. Despite these transcriptional changes, CBD treatment did not significantly alter BAG3, Bcl-2, Cyclin B1, p21, or p53 protein expression in FG0 cells (Figs. 3B–F). In HT1080 fibrosarcoma cells, CBD treatment resulted in a marked reduction in *BAG3* mRNA expression (approximately 11-fold, *p* < 0.01) and a significant decrease in *CCNB1* mRNA levels (approximately 2.5-fold, *p* < 0.05), whereas *CDKN1A (p21)* mRNA expression was significantly increased (Fig. 3G). Although *BCL2* and *TP53* (*p53)* transcript levels showed downward trends following CBD treatment, neither showed a statistically significant change.

At the protein level, CBD treatment significantly reduced the expression of BAG3, Cyclin B1, and p21, while significantly increasing Bcl-2 expression (Figs. 3H-L). No significant changes in p53 protein expression were detected.

These findings demonstrate that CBD selectively modulates apoptosis- and cell-cycle-associated proteins in HT1080 fibrosarcoma cells, whereas comparable transcriptional changes in FG0 cells are not accompanied by corresponding alterations in protein expression.

### CBD selectively increases apoptotic cell populations in HT1080 cells

**Fig. 4:**
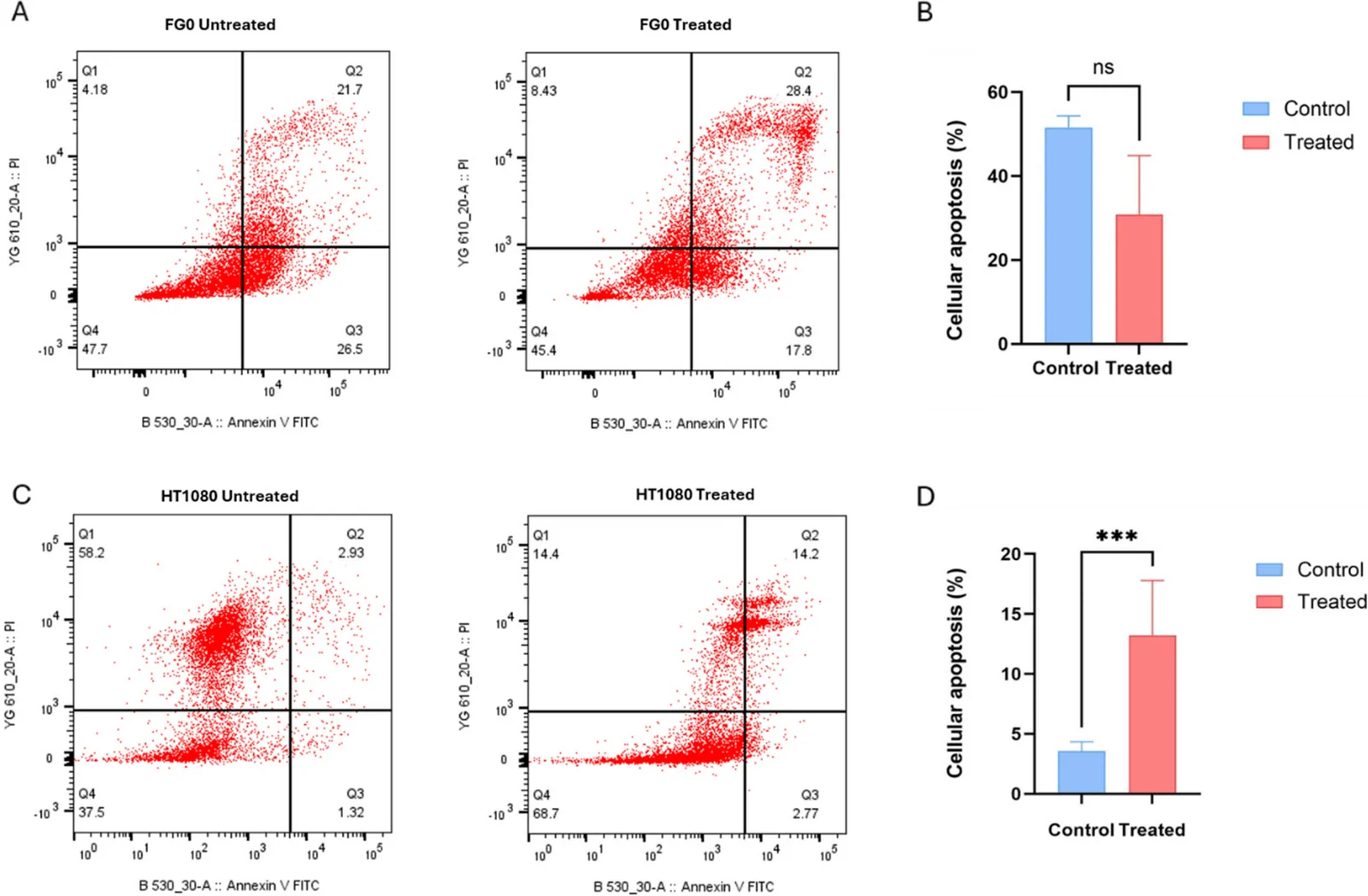
CBD-induced apoptosis in non-cancerous fibroblast (FG0) and fibrosarcoma (HT1080) cells assessed by Annexin V–FITC/PI flow cytometry. **A** Representative flow cytometry dot plots of untreated (Control) and CBD-treated FG0 cells. **B** Quantitative analysis of Annexin V-positive apoptotic FG0 cells. **C** Representative flow cytometry dot plots of untreated (Control) and CBD-treated HT1080 cells. **D** Quantitative analysis of Annexin V-positive apoptotic HT1080 cells. Cell populations were classified as viable (Annexin V⁻/PI⁻; Q4), early apoptotic (Annexin V⁺/PI⁻; Q3), late apoptotic (Annexin V⁺/PI⁺; Q2), and necrotic (Annexin V⁻/PI⁺; Q1). Representative flow cytometry dot plots are shown from one of three independent biological experiments, and quantitative data are presented as mean ± SD from all three independent biological experiments. Statistical significance is indicated as *p* < 0.05 (*), *p* < 0.01 (**), and *p* < 0.001 or *p* < 0.0001 (***)

Annexin V FITC/PI flow cytometric analysis was performed to quantify apoptosis following CBD treatment (Fig. 4). In FG0 cells, CBD treatment did not significantly alter the proportion of Annexin V-positive apoptotic cells (Fig. 4A and B). Although the proportion of apoptotic cells decreased from 51.5% ± 2.84% in untreated controls to 30.8% ± 14.2% following CBD treatment, representing a reduction of 20.7% points, this difference was not statistically significant. These findings indicate that CBD did not significantly alter apoptosis in FG0 cells under the experimental conditions examined.

In contrast, CBD treatment significantly increased apoptosis in HT1080 cells (Fig. 4C and D). The proportion of Annexin V-positive apoptotic cells increased from 3.55% ± 0.78% in untreated controls to 13.2% ± 4.56% following CBD treatment, representing a 9.65%-point increase. These findings demonstrate that CBD significantly promotes apoptosis in HT1080 fibrosarcoma cells under the experimental conditions examined.

### CBD selectively alters cell-cycle progression in HT1080 cells

**Fig. 5:**
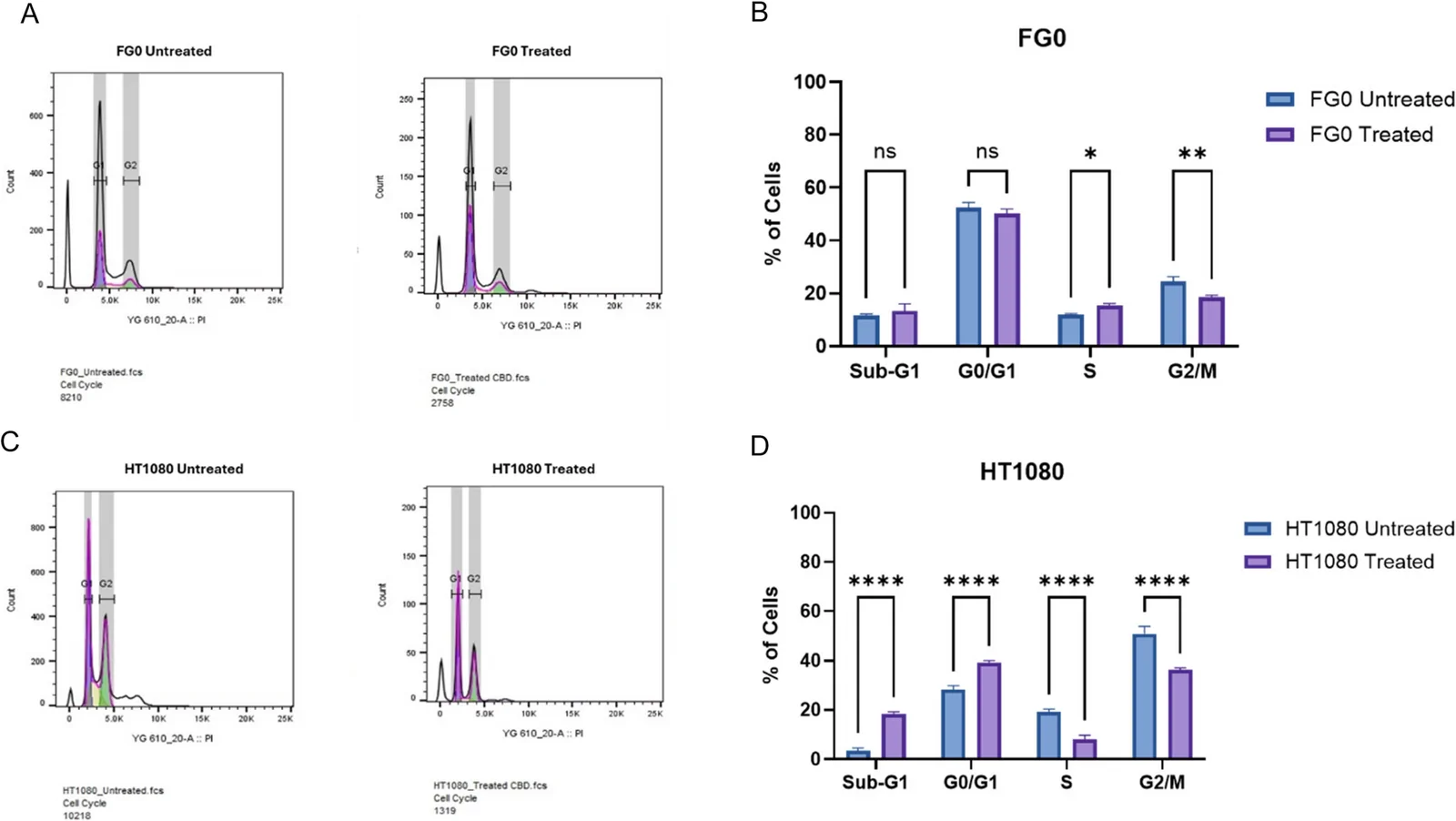
CBD selectively disrupts cell-cycle progression in HT1080 cells while producing only modest changes in FG0 cells. Representative DNA-content histograms generated from propidium iodide (PI) staining and analyzed using the Dean–Jett–Fox modeling algorithm are shown for untreated and CBD-treated FG0 cells (**A**) and HT1080 cells (**C**), with the corresponding quantitative analyses in (**B**) and (**D**), respectively. Cell-cycle distribution was determined by propidium iodide (PI) staining and flow cytometry. The model-derived “% less G1” population was reported as the Sub-G1 fraction, whereas the G2/M fraction was calculated as the sum of the model-derived G2 and “% greater G2” populations. Representative histograms are shown from one of three independent biological experiments, whereas the quantitative data represent the mean ± SD from three independent biological experiments. Cell-cycle distributions were also analyzed independently by manual gating, and the corresponding results are presented in Supplementary Figure S2. Statistical analysis was performed using two-way ANOVA, followed by Šídák’s post hoc multiple comparisons test, to compare the untreated and CBD-treated groups within each cell-cycle phase. Statistical significance is indicated as *p* < 0.05 (*), *p* < 0.01 (**), and *p* < 0.001 or *p* < 0.0001 (***)

Cell-cycle distribution was assessed by propidium iodide (PI) staining and flow cytometry using the Dean–Jett–Fox modeling algorithm (Fig. 5). Cell-cycle distributions were also independently analyzed by manual gating, which showed comparable overall trends (Supplementary Figure S2).

In FG0 cells, CBD treatment did not significantly alter the proportions of cells in the Sub-G1 or G0/G1 populations (Fig. 5A and B). Although the S-phase population increased significantly from 11.90 ± 0.45% in untreated controls to 15.43 ± 0.68% following CBD treatment, with a corresponding decrease in the G2/M population from 24.42 ± 1.90% to 18.59 ± 0.67%, these changes were modest and were not accompanied by significant alterations in either the Sub-G1 or G0/G1 fractions, indicating that CBD did not substantially disrupt overall cell-cycle progression in FG0 cells under the experimental conditions examined.

In contrast, CBD treatment markedly altered cell-cycle distribution in HT1080 cells (Fig. 5C and D). The proportion of cells in the Sub-G1 population increased significantly from 3.44 ± 1.10% (mean ± SD) in untreated controls to 18.37 ± 0.83% following CBD treatment. Similarly, the G0/G1 population increased significantly from 28.30 ± 1.57% to 39.17 ± 0.85%. These changes were accompanied by significant reductions in the S-phase population (19.32 ± 1.07% to 8.08 ± 1.64%) and the G2/M population (50.97 ± 2.87% to 36.17 ± 0.89%). The increase in the Sub-G1 fraction is consistent with the induction of apoptosis observed by Annexin V-FITC/PI analysis (Fig. 4), whereas the accumulation of cells in the G0/G1 phase together with the reduction in the S- and G2/M-phase populations suggests impaired cell-cycle progression following CBD treatment.

## Discussion

### CBD selectively suppresses FAM111B protein expression in fibrosarcoma cells

A key finding of this study is that CBD reduced *FAM111B* mRNA expression in both fibrosarcoma (HT1080) and non-cancerous fibroblast (FG0) cells, whereas a corresponding reduction in FAM111B protein expression is observed only in HT1080 cells. This differential regulation highlights a distinction between transcriptional and post-transcriptional responses to CBD and suggests that FAM111B protein abundance may be selectively susceptible to CBD-mediated regulation in the fibrosarcoma cellular context.

In our previous study (Rhoda et al. 2023), fibrosarcoma cell lines (HT1080) exhibited increased FAM111B mRNA expression and significantly elevated FAM111B protein levels compared with normal fibroblast cell lines, implicating FAM111B in sarcoma-associated proliferative and survival pathways. In addition, siRNA-mediated knockdown of FAM111B in HT1080 fibrosarcoma cells significantly reduced cellular proliferation and migration while promoting apoptosis, supporting a functional role for FAM111B in tumor cell survival (Rhoda et al. 2023). The present findings extend these observations by demonstrating that CBD suppresses FAM111B protein expression while inducing molecular and cellular changes that are broadly consistent with previous FAM111B loss-of-function studies. However, the present study did not directly investigate whether FAM111B suppression is required for the downstream effects of CBD, and therefore, causality cannot be established from the current data.

CBD has been reported to enhance p53 activity in certain cancer models (Wang et al. 2023); however, in the present study, p53 expression remained unchanged at both the mRNA and protein levels following CBD treatment. These findings indicate that CBD-mediated suppression of FAM111B was not accompanied by detectable changes in total p53 mRNA or protein levels, suggesting that alternative regulatory mechanisms may be involved. CBD has been reported to modulate the PI3K/Akt signaling pathway (Franco-Vadillo et al. 2021), which has previously been implicated in FAM111B-driven tumor growth and survival (Yang et al. 2024). Inhibition of this pathway by CBD (Yang et al. 2024) may therefore contribute to reduced FAM111B protein abundance in fibrosarcoma cells.

In addition, CBD has been shown to induce oxidative stress selectively in cancer cells, leading to altered protein stability and degradation (Mangal et al. 2021). Given evidence that FAM111B supports cancer cell survival under conditions of cellular stress (Gong et al. 2022), CBD-induced oxidative stress may preferentially compromise FAM111B protein stability in fibrosarcoma cells. CBD has also been reported to influence epigenetic regulators, including DNA methyltransferase 3B (DNMT3B)(Fuchs Weizman et al. 2021, Gagliardi et al. 2018), and FAM111B expression has been shown to be repressed by DNMT3B-mediated mechanisms under certain conditions (Zhu et al. 2022). While epigenetic regulation was not directly assessed in this study, these pathways warrant further investigation.

### CBD promotes apoptosis in fibrosarcoma cells through disruption of BAG3-associated survival pathways

CBD treatment resulted in a significant increase in apoptotic cell populations in HT1080 fibrosarcoma cells, whereas no increase in apoptosis was observed in non-cancerous fibroblasts used in this study. This functional divergence occurred despite similar sensitivity to CBD in terms of cellular metabolic activity, suggesting that downstream signaling responses, rather than differences in initial cellular exposure, may determine susceptibility to CBD-induced apoptosis. These findings indicate that the cancer-specific effects observed in HT1080 cells are more likely attributable to differential stress-response and survival signaling pathways than to preferential cytotoxic activity of CBD.

A major molecular change associated with apoptosis in fibrosarcoma cells was the pronounced downregulation of BAG3 at both the mRNA and protein levels. BAG3 is frequently overexpressed in chemo-resistant cancers and functions as a critical regulator of cellular stress adaptation by stabilizing anti-apoptotic proteins and chaperone-mediated survival pathways (Das et al. 2018). Through interactions with proteins such as Bcl-2 and Hsp70, BAG3 has been shown to enhance resistance to apoptosis and support tumor cell survival under conditions of cellular stress (Rosati et al. 2011).

Bcl-2 suppresses apoptosis by preventing the activation of the pro-apoptotic proteins BAX and BAK, thereby inhibiting mitochondrial outer membrane permeabilization and cytochrome c release (Brunelle and Letai 2009, Dewson and Kluck 2009, Dlugosz et al. 2006, Shamas-Din et al. 2013). Hsp70 further supports this process by inhibiting BAX activation and stabilizing Bcl2-dependent signaling (Ruchalski et al. 2006, Jäättelä et al. 1998). Interestingly, Bcl-2 protein levels were increased following CBD treatment in fibrosarcoma cells despite the induction of apoptosis. This apparent paradox may reflect a compensatory cellular response to stress. However, the concurrent loss of BAG3 may compromise Bcl-2 function and the ability of its associated chaperone networks, thereby lowering the threshold for apoptosis. However, the present study did not assess downstream apoptotic mediators such as caspase activation or BAX/BAK signaling, and therefore, the precise mechanisms underlying the coexistence of increased Bcl-2 expression and enhanced apoptosis remain to be determined.

Several additional factors may contribute to the pro-apoptotic effects of CBD. CBD has been reported to influence mitochondrial function through interactions with voltage-dependent anion channel 1 (VDAC1), leading to altered calcium homeostasis, impaired metabolite transport, and the promotion of cytochrome c release (Olivas-Aguirre et al. 2019, Rimmerman et al. 2013). CBD has also been shown to inhibit pro-survival signaling pathways, including PI3K/Akt, thereby suppressing anti-apoptotic mediators and activating apoptotic signaling cascades (Chen et al. 2021). Furthermore, CBD-induced oxidative stress and mitochondrial dysfunction have been linked to activation of caspase-dependent apoptosis in multiple cancer models (Ma et al. 2023, Shrivastava et al. 2011). Collectively, these mechanisms provide a plausible explanation for the selective induction of apoptosis observed in fibrosarcoma cells following CBD treatment.

BAG3 has also been implicated in regulating autophagy by stabilizing glutaminase (GLS) and promoting glutaminolysis (Zhao et al. 2019). Disruption of autophagy can sensitize cancer cells to apoptosis (Liu et al. 2023), and inhibition of autophagic pathways has been shown to enhance apoptotic cell death in liver cancer models (Li et al. 2020). Although autophagy was not directly assessed in this study, BAG3 downregulation may contribute to impaired stress adaptation and apoptotic susceptibility to apoptosis in CBD-treated fibrosarcoma cells.

While the present findings demonstrate an association between CBD-mediated FAM111B suppression, BAG3 downregulation, and apoptosis, direct mechanistic interactions between these pathways were not investigated. Consequently, it remains unclear whether FAM111B functions as a primary mediator of the observed apoptotic response or represents a downstream component of a broader stress-response network activated by CBD. Therefore, additional studies will be required to determine whether BAG3 regulation occurs downstream of FAM111B suppression or represents an independent component of the cellular response to CBD.

### CBD selectively disrupts cell-cycle progresxxsion in fibrosarcoma cells

CBD treatment significantly altered cell-cycle distribution in HT1080 fibrosarcoma cells, characterized by increased Sub-G1 and G0/G1 populations together with reductions in the S- and G2/M-phase populations. In contrast, CBD produced only modest changes in S- and G2/M-phase distributions in non-cancerous FG0 fibroblasts, without significant alterations in the Sub-G1 or G0/G1 populations. The marked increase in the Sub-G1 fraction in HT1080 cells is consistent with the induction of apoptosis observed by Annexin V-FITC/PI analysis, suggesting that CBD disrupts cell-cycle progression and promotes apoptotic cell death in fibrosarcoma cells.

One of the principal molecular changes associated with these alterations was the significant reduction in Cyclin B1 expression at both the mRNA and protein levels. Cyclin B1 forms a regulatory complex with cyclin-dependent kinase 1 (CDK1) that is essential for progression through the G2/M transition and entry into mitosis (Porter and Donoghue 2003, Lindqvist et al. 2007, Gavet and Pines 2010). Its downregulation is therefore consistent with impaired cell cycle progression and entry into a quiescent state (Cooper et al. 2007), and may contribute to the reduced proportion of cells progressing through the S and G2/M phases following CBD treatment.

CBD treatment also increased *CDKN1A (p21)* mRNA expression while reducing p21 protein abundance in HT1080 cells. p21 is a cyclin-dependent kinase inhibitor that regulates cell-cycle progression through multiple checkpoints, including the G1/S and G2/M transitions (Kreis et al. 2019, Abbas and Dutta 2009). Previous studies have shown that CBD can activate stress-responsive signaling pathways that promote p21 transcription (Jastrząb et al. 2019, Jeong et al. 2019). However, oxidative stress and altered protein turnover may destabilize p21 protein or influence its intracellular localization, resulting in discordance between transcript and protein abundance (Abbas and Dutta 2009, Bendjennat et al. 2003). The present findings therefore suggest that post-transcriptional or post-translational regulatory mechanisms contribute to p21 regulation following CBD exposure, although these mechanisms were not investigated directly.

Unlike Cyclin B1 and p21, total p53 mRNA and protein expression remained unchanged following CBD treatment. These findings indicate that the observed alterations in cell-cycle progression occurred in the absence of detectable changes in total p53 expression under the experimental conditions examined. However, given that p53 activity is highly dynamic and is frequently regulated through post-translational modifications, protein stabilization, and transient activation rather than changes in total expression (Che et al. 2020, Liu et al. 2019, Luo et al. 2017). Consequently, the present study does not exclude a role for functional p53 signaling, and additional studies examining p53 activation status and temporal responses will be required to clarify its contribution to CBD-mediated cell-cycle regulation.

Thus, these findings suggest that CBD selectively disrupts cell-cycle progression in HT1080 fibrosarcoma cells while exerting comparatively limited effects on non-cancerous fibroblasts. Together with the selective reduction in FAM111B protein expression, BAG3 suppression, and increased apoptosis observed in the present study, these findings support a mechanistic model in which CBD compromises stress-adaptive and proliferative pathways utilized by fibrosarcoma cells.

### Study limitations, implications, and future directions

A limitation of the present study is that cellular metabolic activity was assessed using the MTT assay, which reflects mitochondrial metabolic function and does not directly distinguish between cytostatic and cytotoxic effects. Consequently, the reductions in MTT signal observed following CBD treatment should be interpreted as evidence of reduced cellular metabolic activity rather than as definitive evidence of cell death. In addition, the present study focused on early molecular and cellular responses following 24 h of CBD exposure and evaluated downstream molecular effects at concentrations approximating the experimentally determined IC₅₀ values. While this approach enabled mechanistic investigation under biologically relevant experimental conditions, it did not permit assessment of response kinetics, signaling thresholds, or adaptive cellular responses that may emerge during prolonged exposure. Future studies employing broader dose-response analyses, extended treatment time courses, and clonogenic survival assays will be necessary to further characterize the pharmacodynamic effects of CBD in fibrosarcoma models. Furthermore, the concentrations of CBD used in this study are generally higher than those typically reported in human plasma following standard clinical dosing and should therefore be interpreted as mechanistic concentrations for in vitro investigation rather than direct indicators of clinically achievable therapeutic efficacy. Accordingly, the present findings provide mechanistic evidence that CBD modulates stress, survival, and cell-cycle pathways in fibrosarcoma cells rather than as direct predictors of in vivo antitumor efficacy.

An additional limitation of this study is the use of a single fibrosarcoma cell line (HT1080). Although HT1080 cells are widely used as a fibrosarcoma model, their unique genetic and metabolic characteristics may limit the generalizability of the present findings. Validation in additional fibrosarcoma and sarcoma models will therefore be necessary. Furthermore, the present study utilized conventional two-dimensional cell culture systems as an initial proof-of-concept platform to investigate CBD-mediated molecular responses. While these models are useful for mechanistic investigations, they do not fully recapitulate the complexity of the tumor microenvironment or the pharmacokinetic factors that influence CBD activity in vivo. Consequently, future studies employing more physiologically relevant experimental models will be required to validate and extend the present findings.

Taken together, these findings demonstrate that CBD does not act as a broadly selective cytotoxic agent in this experimental system but rather functions as a context-dependent modulator of stress, survival, and cell-cycle pathways in HT1080 fibrosarcoma cells. The selective reduction in FAM111B protein expression, suppression of BAG3, induction of apoptosis, and distruption of cell-cycle progression define a molecular response that distinguishes HT1080 fibrosarcoma cells from non-cancerous fibroblasts following CBD treatment.

Future studies should investigate the mechanisms underlying FAM111B protein regulation, including proteasomal degradation, autophagy, and epigenetic control. While previous work from our group demonstrated that FAM111B knockdown promotes apoptosis and suppresses malignant phenotypes in HT1080 fibrosarcoma cells (Rhoda et al. 2023), additional studies employing FAM111B knockdown, overexpression, and rescue approaches in independent fibrosarcoma and other sarcoma models will be required to determine whether FAM111B directly mediates the effects of CBD on apoptosis, BAG3 regulation, and cell-cycle progression. Further work should also evaluate the therapeutic potential of combining CBD with agents that disrupt stress-adaptive survival pathways in sarcoma. Finally, studies using clinically relevant exposure conditions and in vivo models will be necessary to establish the broader biological and translational significance of these findings.

## Conclusion

This study demonstrates that CBD modulates key molecular pathways associated with fibrosarcoma cell survival and proliferation in a context-dependent manner. CBD reduced *FAM111B* mRNA expression in both fibrosarcoma and non-cancerous fibroblast cells, while selectively decreasing FAM111B protein expression in fibrosarcoma cells. This selective protein-level regulation was accompanied by suppression of BAG3, induction of apoptosis, and disruption of cell-cycle progression characterized by increased Sub-G1 and G0/G1 populations together with reduced S- and G2/M-phase populations.

Importantly, these responses occurred in the absence of detectable changes in total p53 mRNA or protein expression and were not observed to the same extent in non-cancerous fibroblasts, highlighting a context-dependent cellular response to CBD. Together, these findings identify FAM111B- and BAG3-associated pathways as potential vulnerabilities in HT1080 fibrosarcoma cells and support further investigation of CBD as a mechanistic modulator of stress adaptation, apoptosis and cell-cycle regulation in preclinical sarcoma models. Future studies will be required to determine whether FAM111B directly mediates these effects and to establish their relevance in additional preclinical sarcoma models.

## Supplementary Information

Supplementary Material 1.

Supplementary Material 2.

## Data Availability

The data generated in this study are available from the corresponding author upon reasonable request.

## References

[CR53] Abbas T, Dutta A. p21 in cancer: intricate networks and multiple activities. Nat Rev Cancer. 2009;9(6):400–14. 10.1038/nrc2657 PMC2722839 19440234

[CR17] Arowolo A, Malebana M, Sunda F, Rhoda C. Proposed Cellular Function of the Human FAM111B Protein and Dysregulation in Fibrosis and Cancer. Front Oncol. 2022;12:932167. 10.3389/fonc.2022.932167 PMC9293052 35860584

[CR3] Augsburger D, Nelson PJ, Kalinski T, Udelnow A, Knösel T, Hofstetter M, et al. Current diagnostics and treatment of fibrosarcoma -perspectives for future therapeutic targets and strategies. Oncotarget. 2017;8(61):104638–53. 10.18632/oncotarget.20136 PMC5732833 29262667

[CR4] Bansal A, Goyal S, Goyal A, Jana M. WHO classification of soft tissue tumours 2020: an update and simplified approach for radiologists. Eur J Radiol. 2021;143:109937. 10.1016/j.ejrad.2021.109937 34547634

[CR56] Bendjennat M, Boulaire J, Jascur T, Brickner H, Barbier V, Sarasin A, et al. UV irradiation triggers ubiquitin-dependent degradation of p21WAF1 to promote DNA repair. Cell. 2003;114(5):599–610. 10.1016/j.cell.2003.08.001 13678583

[CR34] Brunelle JK, Letai A. Control of mitochondrial apoptosis by the Bcl-2 family. J Cell Sci. 2009;122(4):437–41. 10.1242/jcs.031682 PMC2714431 19193868

[CR57] Che Z, Sun H, Yao W, Lu B, Han Q. Role of post-translational modifications in regulation of tumor suppressor p53 function. Front Oral Maxillofacial Med. 2020;2:1–11.

[CR42] Chen S, Deng C, Zheng W, Li S, Liu Y, Zhang T, et al. Cannabidiol effectively promoted cell death in bladder cancer and the improved intravesical adhesion drugs delivery strategy could be better used for treatment. Pharmaceutics. 2021;13(9):1415. 10.3390/pharmaceutics13091415 PMC8471856 34575494

[CR51] Cooper S, Paulsen M, Ljungman M, Vu-Phan D, Kim D, Gonzalez-Hernandez M. Membrane-elution analysis of content of cyclins A, B1, and E during the unperturbed mammalian cell cycle. Cell Div. 2007;2(1):28. 10.1186/1747-1028-2-28 PMC2064908 17892542

[CR32] Das CK, Linder B, Bonn F, Rothweiler F, Dikic I, Michaelis M, et al. BAG3 overexpression and cytoprotective autophagy mediate apoptosis resistance in chemoresistant breast cancer cells. Neoplasia. 2018;20(3):263–79. 10.1016/j.neo.2018.01.001 PMC5852393 29462756

[CR35] Dewson G, Kluck RM. Mechanisms by which Bak and Bax permeabilise mitochondria during apoptosis. J Cell Sci. 2009;122(16):2801–8. 10.1242/jcs.038166 PMC2736138 19795525

[CR36] Dlugosz PJ, Billen LP, Annis MG, Zhu W, Zhang Z, Lin J, et al. Bcl-2 changes conformation to inhibit Bax oligomerization. EMBO J. 2006;25(11):2287–96. 10.1038/sj.emboj.7601126 PMC1478188 16642033

[CR1] Ferreira CR, da Fonseca LG, Piotto GHM, Geyer FC, de Alcântara PSM. Fibrosarcoma: a challenging diagnosis. Autopsy Case Rep. 2013;3(3):21. 10.4322/acr.2013.024 PMC6671889 31528615

[CR25] Franco-Vadillo A, Toledo-Blass M, Rivera-Herrera Z, Guevara-Balcazar G, Orihuela-Rodriguez O, Morales-Carmona JA, et al. Cannabidiol-mediated RISK PI3K/AKT and MAPK/ERK pathways decreasing reperfusion myocardial damage. Pharmacol Res Perspect. 2021;9(4):e00784. 10.1002/prp2.784 PMC8236079 34176244

[CR29] Fuchs Weizman N, Wyse BA, Szaraz P, Defer M, Jahangiri S, Librach CL. Cannabis alters epigenetic integrity and endocannabinoid signalling in the human follicular niche. Hum Reprod. 2021;36(7):1922–31. 10.1093/humrep/deab104 PMC8213445 33954787

[CR30] Gagliardi M, Strazzullo M, Matarazzo MR. DNMT3B functions: novel insights from human disease. Front cell Dev biology. 2018;6:140. 10.3389/fcell.2018.00140 PMC6204409 30406101

[CR50] Gavet O, Pines J. Progressive activation of CyclinB1-Cdk1 coordinates entry to mitosis. Dev Cell. 2010;18(4):533–43. 10.1016/j.devcel.2010.02.013 PMC3325599 20412769

[CR6] Gómez-Puerto D, Cicala CM, Valverde C, Serrano C. Emerging treatments for sarcoma: from 2024 onward. Expert Opin Emerg Drugs. 2025;30(1):53–70. 10.1080/14728214.2025.2460525 39874592

[CR28] Gong Q, Dong Q, Zhong B, Zhang T, Cao D, Zhang Y, et al. Clinicopathological features, prognostic significance, and associated tumor cell functions of family with sequence similarity 111 member B in pancreatic adenocarcinoma. J Clin Lab Anal. 2022;36(12):e24784. 10.1002/jcla.24784 PMC9756984 36408702

[CR12] Hoffmann S, Pentakota S, Mund A, Haahr P, Coscia F, Gallo M, et al. FAM111 protease activity undermines cellular fitness and is amplified by gain-of‐function mutations in human disease. EMBO Rep. 2020;21(10):e50662. 10.15252/embr.202050662 PMC7534640 32776417

[CR39] Jäättelä M, Wissing D, Kokholm K, Kallunki T, Egeblad M. Hsp70 exerts its anti-apoptotic function downstream of caspase‐3‐like proteases. EMBO J. 1998;17(21):6124–34. 10.1093/emboj/17.21.6124 PMC1170939 9799222

[CR54] Jastrząb A, Gęgotek A, Skrzydlewska E. Cannabidiol regulates the expression of keratinocyte proteins involved in the inflammation process through transcriptional regulation. Cells. 2019;8(8):827. 10.3390/cells8080827 PMC6721680 31382646

[CR55] Jeong S, Yun HK, Jeong YA, Jo MJ, Kang SH, Kim JL, et al. Cannabidiol-induced apoptosis is mediated by activation of Noxa in human colorectal cancer cells. Cancer Lett. 2019;447:12–23. 10.1016/j.canlet.2019.01.011 30660647

[CR52] Kreis N-N, Louwen F, Yuan J. The multifaceted p21 (Cip1/Waf1/CDKN1A) in cell differentiation, migration and cancer therapy. Cancers. 2019;11(9):1220. 10.3390/cancers11091220 PMC6770903 31438587

[CR22] Kose T. A Comparison of GAPDH and ACTB As Internal Control for Gene Expression Studies in Different Cancer Cell Lines. UHOD: Int J Hematol Oncology/Uluslararasi Hematoloji Onkoloji Dergisi. 2024;34(3):140–7.

[CR47] Li Y, Zhu Y, Fang J, Li W, Li S, Liu X, et al. Apoptin regulates apoptosis and autophagy by modulating reactive oxygen species (ROS) levels in human liver cancer cells. Front Oncol. 2020;10:1026. 10.3389/fonc.2020.01026 PMC7344208 32714864

[CR49] Lindqvist A, Van Zon W, Karlsson Rosenthal C, Wolthuis RMF. Cyclin B1–Cdk1 activation continues after centrosome separation to control mitotic progression. PLoS Biol. 2007;5(5):e123. 10.1371/journal.pbio.0050123 PMC1858714 17472438

[CR58] Liu Y, Tavana O, Gu W. p53 modifications: exquisite decorations of the powerful guardian. J Mol Cell Biol. 2019;11(7):564–77. 10.1093/jmcb/mjz060 PMC6736412 31282934

[CR46] Liu S, Yao S, Yang H, Liu S, Wang Y, Autophagy. Regulator of cell death. Cell Death Dis. 2023;14(10):648. 10.1038/s41419-023-06154-8 PMC10551038 37794028

[CR23] Livak KJ, Schmittgen TD. Analysis of relative gene expression data using real-time quantitative PCR and the 2 – ∆∆CT method. Methods. 2001;25(4):402–8. 10.1006/meth.2001.1262 11846609

[CR59] Luo Q, Beaver JM, Liu Y, Zhang Z. Dynamics of p53: a master decider of cell fate. Genes. 2017;8(2):66.

[CR43] Ma L, Zhang H, Liu C, Liu M, Shangguan F, Liu Y, et al. A novel mechanism of cannabidiol in suppressing ovarian cancer through LAIR-1 mediated mitochondrial dysfunction and apoptosis. Environ Toxicol. 2023;38(5):1118–32. 10.1002/tox.23752 36810933

[CR20] Ma L, Liu M, Liu C, Zhang H, Yang S, An J, et al. Research progress on the mechanism of the antitumor effects of Cannabidiol. Molecules. 2024;29(9):1943. 10.3390/molecules29091943 PMC11085351 38731434

[CR27] Mangal N, Erridge S, Habib N, Sadanandam A, Reebye V, Sodergren MH. Cannabinoids in the landscape of cancer. J Cancer Res Clin Oncol. 2021;147(9):2507–34. 10.1007/s00432-021-03710-7 PMC8310855 34259916

[CR18] Naicker D, Rhoda C, Sunda F, Arowolo A. Unravelling the Intricate Roles of FAM111A and FAM111B: From Protease-Mediated Cellular Processes to Disease Implications. Int J Mol Sci. 2024;25(5):2845. 10.3390/ijms25052845 PMC10931937 38474092

[CR40] Olivas-Aguirre M, Torres-López L, Valle-Reyes JS, Hernández-Cruz A, Pottosin I, Dobrovinskaya O. Cannabidiol directly targets mitochondria and disturbs calcium homeostasis in acute lymphoblastic leukemia. Cell Death Dis. 2019;10(10):779. 10.1038/s41419-019-2024-0 PMC6791884 31611561

[CR48] Porter LA, Donoghue DJ. Cyclin B1 and CDK1: nuclear localization and upstream regulators. Prog Cell Cycle Res. 2003;5:335–48. 14593728

[CR8] Rhoda C, Sunda F, Kidzeru E, Khumalo NP, Arowolo A. FAM111B dysregulation promotes malignancy in fibrosarcoma and POIKTMP and a low-cost method for its mutation screening. Cancer Treat Res Commun. 2023;34:100679. 10.1016/j.ctarc.2022.100679 36610347

[CR41] Rimmerman N, Ben-Hail D, Porat Z, Juknat A, Kozela E, Daniels M, et al. Direct modulation of the outer mitochondrial membrane channel, voltage-dependent anion channel 1 (VDAC1) by cannabidiol: a novel mechanism for cannabinoid-induced cell death. Cell Death Dis. 2013;4(12):e949–e. 10.1038/cddis.2013.471 PMC3877544 24309936

[CR33] Rosati A, Graziano V, De Laurenzi V, Pascale M, Turco MC. BAG3: a multifaceted protein that regulates major cell pathways. Cell Death Dis. 2011;2(4):e141–e. 10.1038/cddis.2011.24 PMC3122056 21472004

[CR14] Roversi G, Colombo EA, Magnani I, Gervasini C, Maggiore G, Paradisi M, et al. Spontaneous chromosomal instability in peripheral blood lymphocytes from two molecularly confirmed Italian patients with Hereditary Fibrosis Poikiloderma: insights into cancer predisposition. Genet Mol Biol. 2021;44(3):e20200332. 10.1590/1678-4685-GMB-2020-0332 PMC8345126 34358284

[CR38] Ruchalski K, Mao H, Li Z, Wang Z, Gillers S, Wang Y, et al. Distinct hsp70 domains mediate apoptosis-inducing factor release and nuclear accumulation. J Biol Chem. 2006;281(12):7873–80. 10.1074/jbc.M513728200 16407317

[CR5] Sahin TK, Germetaki T, Guven DC, Akin S, Dizdar O, Thistlethwaite F, et al. Cell therapy in sarcoma: current landscape and future directions. J Immunother Cancer. 2026;14(1):e013396. 10.1136/jitc-2025-013396 PMC12815135 41526168

[CR37] Shamas-Din A, Kale J, Leber B, Andrews DW. Mechanisms of action of Bcl-2 family proteins. Cold Spring Harb Perspect Biol. 2013;5(4):a008714. 10.1101/cshperspect.a008714 PMC3683897 23545417

[CR44] Shrivastava A, Kuzontkoski PM, Groopman JE, Prasad A. Cannabidiol induces programmed cell death in breast cancer cells by coordinating the cross-talk between apoptosis and autophagy. Mol Cancer Ther. 2011;10(7):1161–72. 10.1158/1535-7163.MCT-10-1100 21566064

[CR2] Sultana R, Sailaja S. Adult fibrosarcoma: From clinical challenges to cutting-edge innovations. Oncoscience. 2025;12:91. 10.18632/oncoscience.624 PMC12410976 40917595

[CR7] Sun H, Liu K, Huang J, Sun Q, Shao C, Luo J, et al. FAM111B, a direct target of p53, promotes the malignant process of lung adenocarcinoma. OncoTargets therapy. 2019;12:2829. 10.2147/OTT.S190934 PMC6489872 31114230

[CR19] Sunda F, Arowolo A. A molecular basis for the anti-inflammatory and anti-fibrosis properties of cannabidiol. Faseb j. 2020;34(11):14083–92. 10.1096/fj.202000975R 32885502

[CR11] Tomioka Y, Seki N, Suetsugu T, Hagihara Y, Sanada H, Goto Y, et al. Identification of tumor suppressive miR-144-5p Targets: FAM111B expression accelerates the malignant phenotypes of lung adenocarcinoma. Int J Mol Sci. 2024;25(18):9974. 10.3390/ijms25189974 PMC11432174 39337462

[CR21] Tong S, Loilome W, Namwat N, Klanrit P, Wangwiwatsin A, Win ZZ, et al. Selective anti-cancer effects of cannabidiol and ∆9-tetrahydrocannabinol via PI3K/AKT/mTOR inhibition and PTEN restoration in ovarian cancer cells. Front Pharmacol. 2025;16:1693129. 10.3389/fphar.2025.1693129 PMC12745404 41472794

[CR24] Wang F, Dezfouli AB, Khosravi M, Sievert W, Stangl S, Schwab M, et al. Cannabidiol-induced crosstalk of apoptosis and macroautophagy in colorectal cancer cells involves p53 and Hsp70. Cell death discovery. 2023;9(1):286. 10.1038/s41420-023-01578-9 PMC10403543 37542074

[CR16] Wang W, Gu Y, Ni H, Quan Q, Guo L. Silencing of FAM111B inhibits tumor growth and promotes apoptosis by decreasing AKT activity in ovarian cancer. Experimental Biology Med. 2023;248(12):1043–55. 10.1177/15353702231160326 PMC10581161 37095701

[CR10] Wang H, Zhu J, Wang H, Zheng W, Wang L, Zhu J, et al. The role of FAM111B in the malignant progression and molecular regulation of human glioma through the PI3K/Akt pathway. Chin Neurosurgical J. 2025;11(1):9. 10.1186/s41016-025-00395-6 PMC12087166 40390043

[CR13] Wei F, Li W, Zhou T, Yuan X, Zhang L. Unveiling FAM111B: A Pan-Cancer Biomarker for DNA Repair and Immune Infiltration. Int J Mol Sci. 2025;26(7):3151. 10.3390/ijms26073151 PMC11989846 40243892

[CR9] Wu H, Liang C. Pan-Cancer Analysis of the Tumorigenic Effect and Prognostic Diagnostic Value of FAM111B in Human Carcinomas. Int J Gen Med. 2023;16:1845–65. 10.2147/IJGM.S409690 PMC10199687 37213474

[CR26] Yang J, Lin N, Li S, Dong Z, Wang D, Liu Y, et al. Cannabidiol alleviates oral mucositis by inhibiting PI3K/Akt/NF-κB-mediated pyroptosis. Balkan Med J. 2024;41(4):286. 10.4274/balkanmedj.galenos.2024.2024-2-66 PMC11588905 38966918

[CR15] Yang Y, Yan Z, Jiao Y, Yang W, Cui Q, Chen S. Family with sequence similarity 111 member B contributes to tumor growth and metastasis by mediating cell proliferation, invasion, and EMT via transforming acidic coiled-coil protein 3/PI3K/AKT signaling pathway in hepatocellular carcinoma. Environ Toxicol. 2024;39(1):409–20. 10.1002/tox.23965 37782700

[CR45] Zhao S, Wang J-M, Yan J, Zhang D-L, Liu B-Q, Jiang J-Y, et al. BAG3 promotes autophagy and glutaminolysis via stabilizing glutaminase. Cell Death Dis. 2019;10(4):284. 10.1038/s41419-019-1504-6 PMC6433946 30910998

[CR31] Zhu X, Xue C, Kang X, Jia X, Wang L, Younis MH, et al. DNMT3B-mediated FAM111B methylation promotes papillary thyroid tumor glycolysis, growth and metastasis. Int J Biol Sci. 2022;18(11):4372. 10.7150/ijbs.72397 PMC9295055 35864964

