# Supplementary material for: Cannabidiol selectively downregulates FAM111B expression and induces apoptosis in fibrosarcoma cells: an in vitro study
Source: J Cannabis Res. 2026 Aug 1;8:116. doi: 10.1186/s42238-026-00481-1 (PMC13721555; doi:10.1186/s42238-026-00481-1)

Full Images of western blot

FGO FAM111B (Fig 2 B)

Control Treated

250
130
100
70
55
35

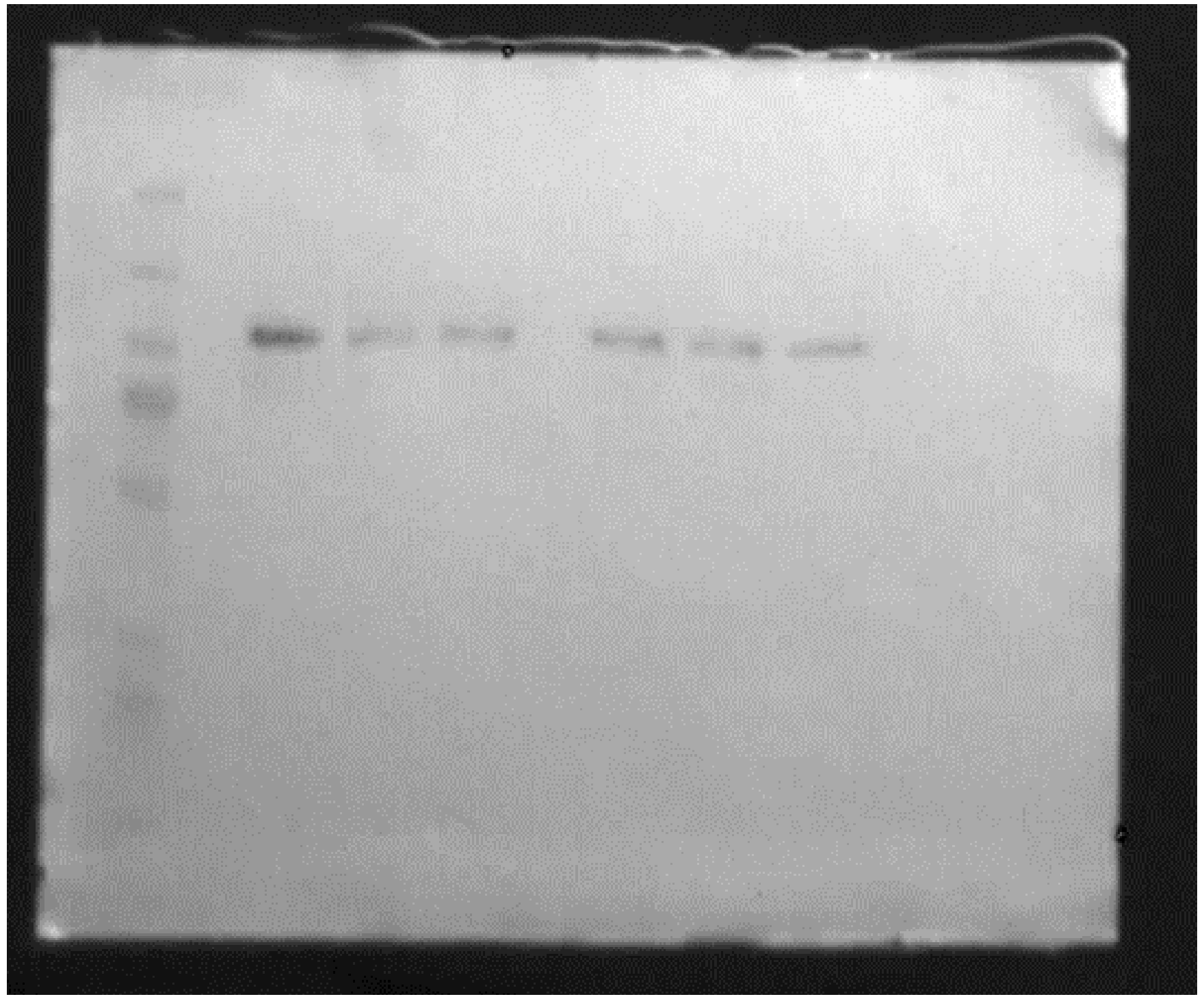

FAM111B
@85KDa

Control Treated

250
130
100
70
55
35

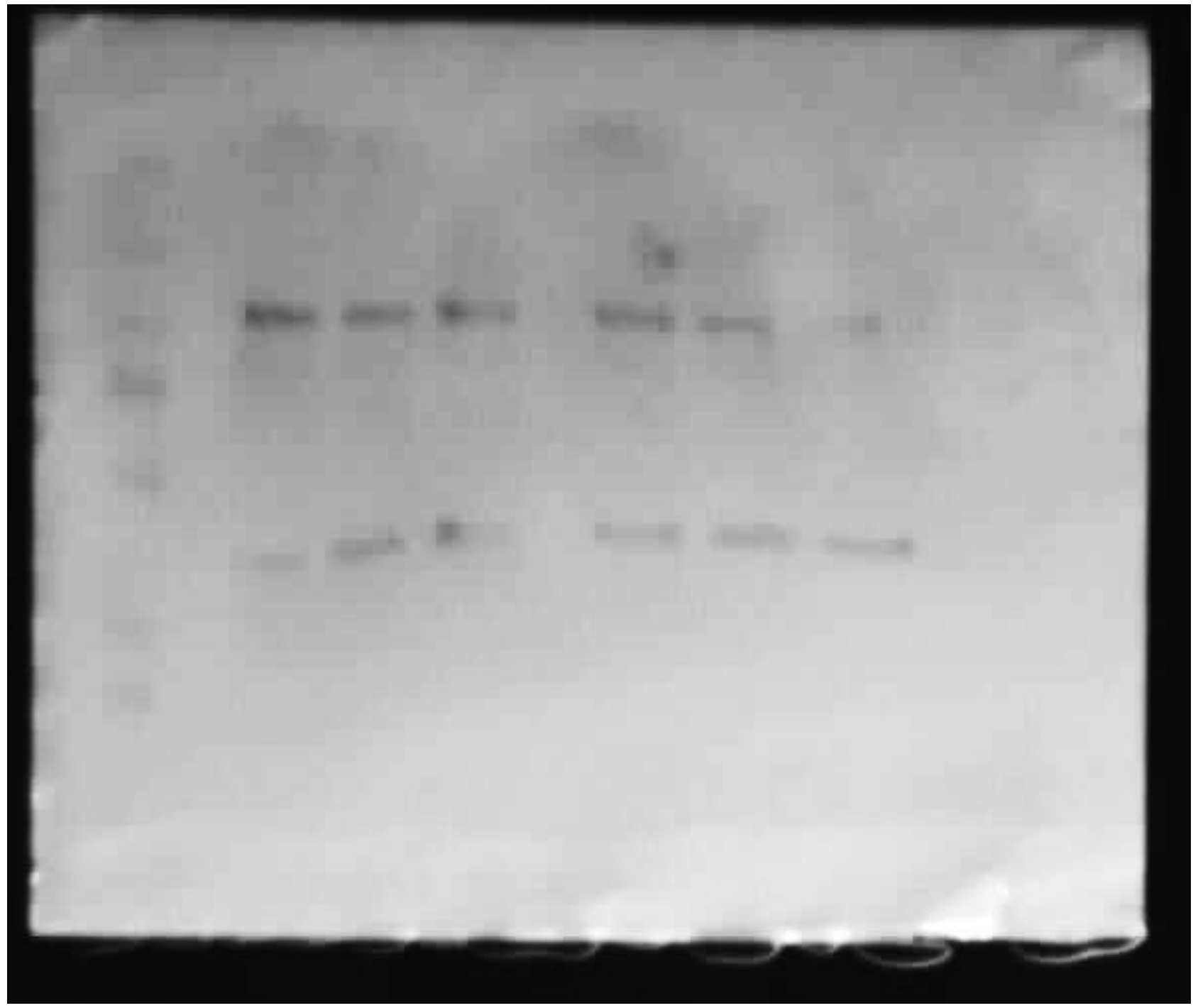

FAM111B @
85KDa

GAPDH @ 36 KDa

HT1080 FAM111B (Fig 2 D)

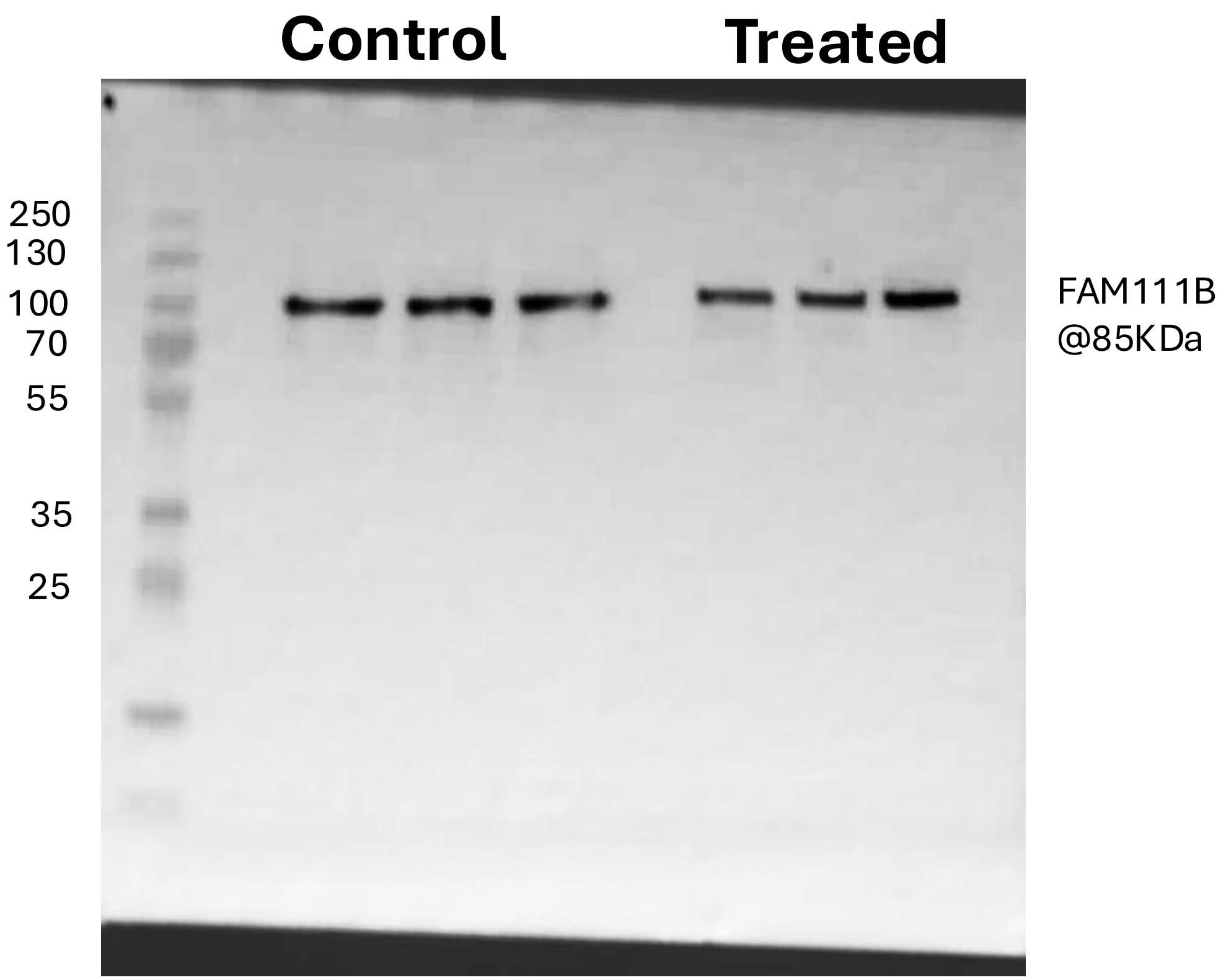

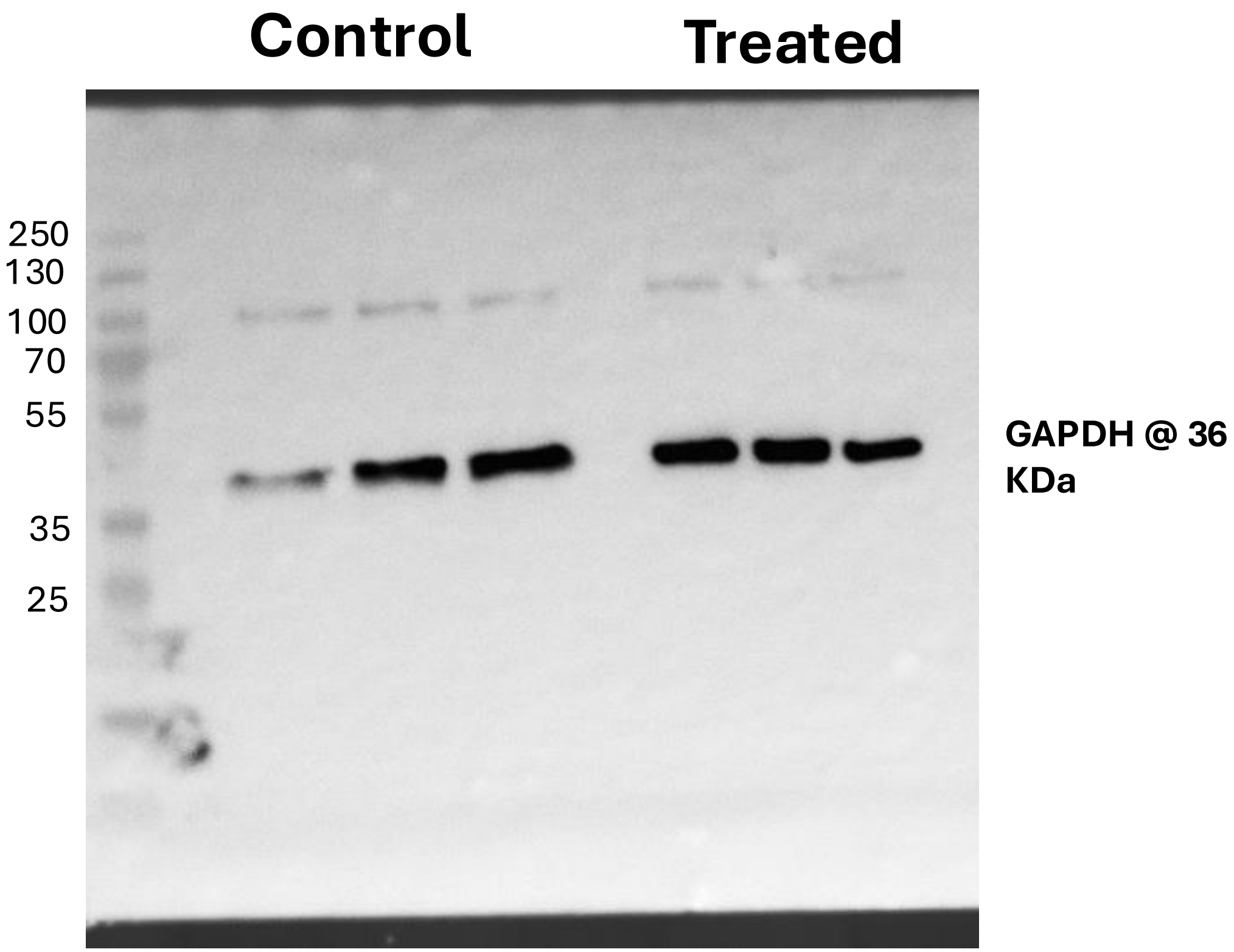

FG0 Cyclin B (Fig 3 B)

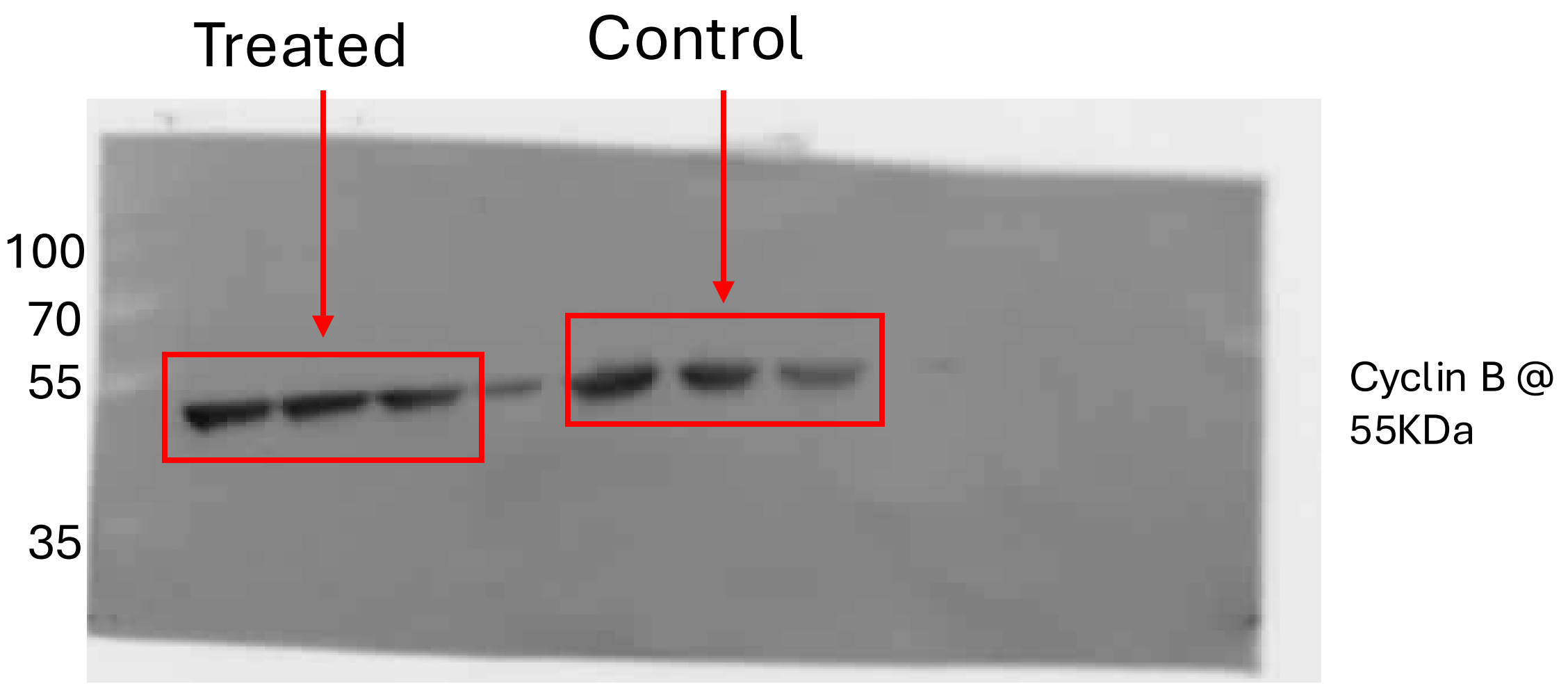

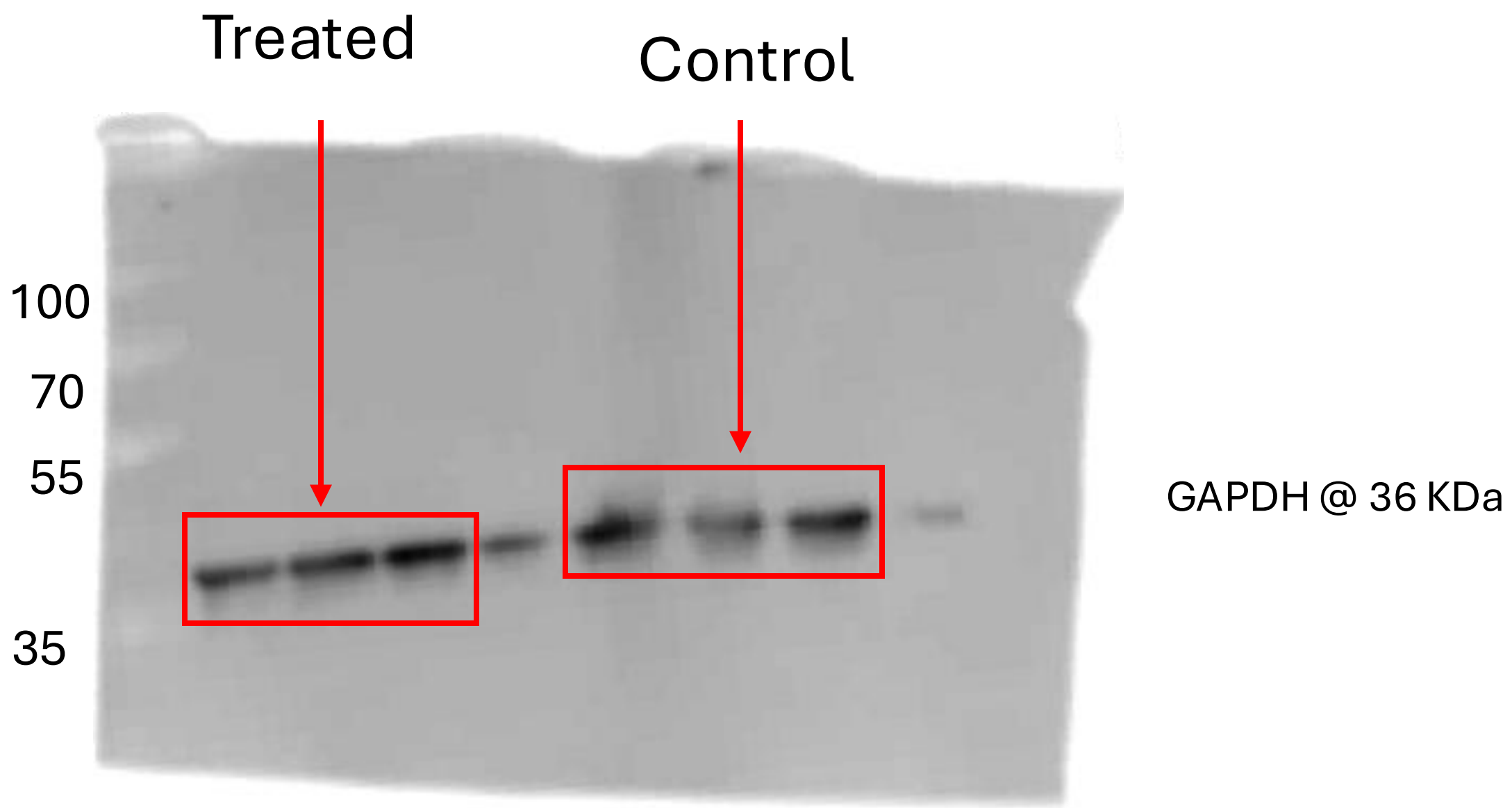

FG0 BAG3 (Fig 3 C)

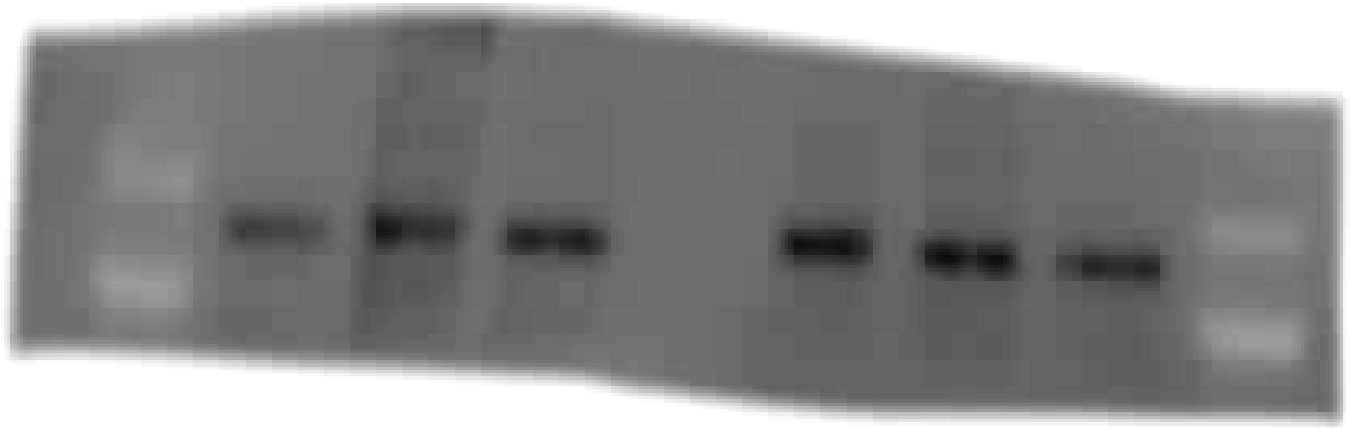

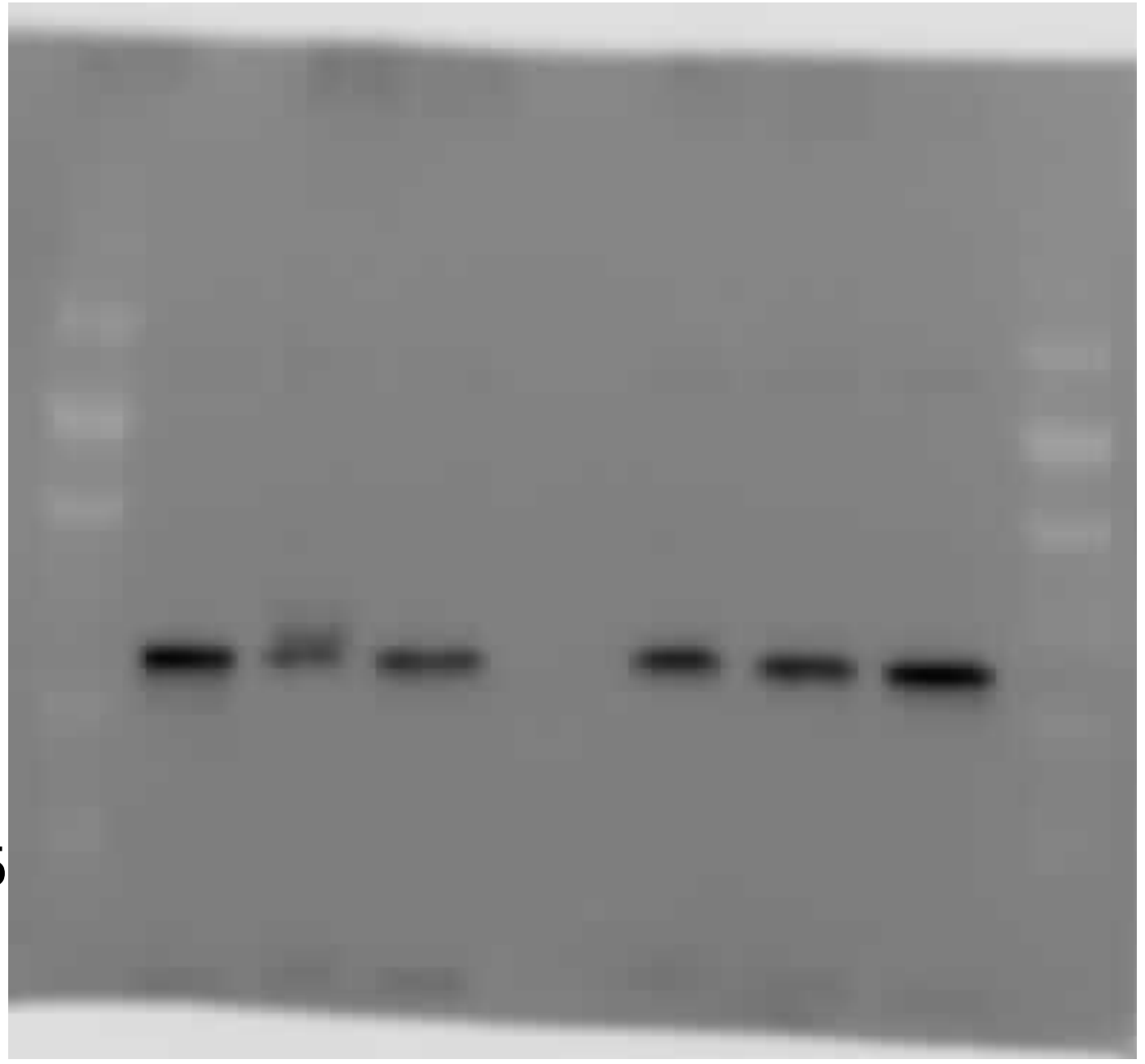

FG0 P21 (Fig 3D)

Control Treated

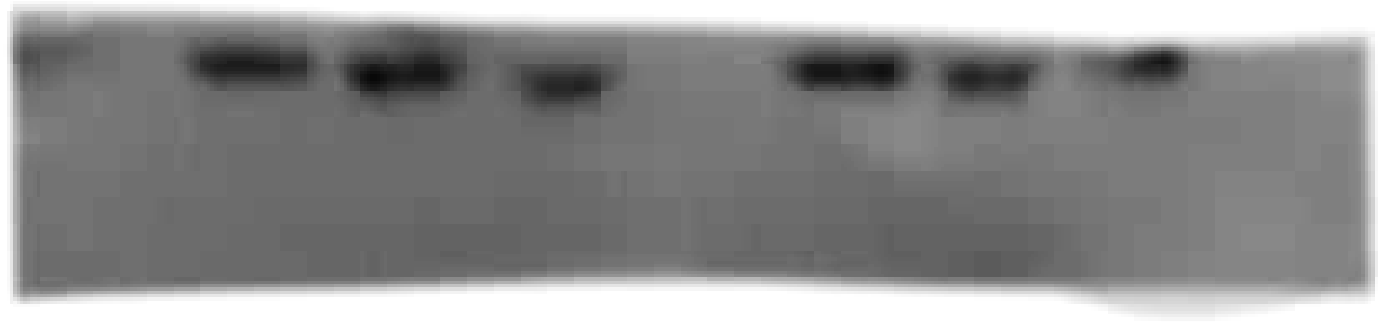

P21 @26kDa

Control Treated

100
70
55
35
25

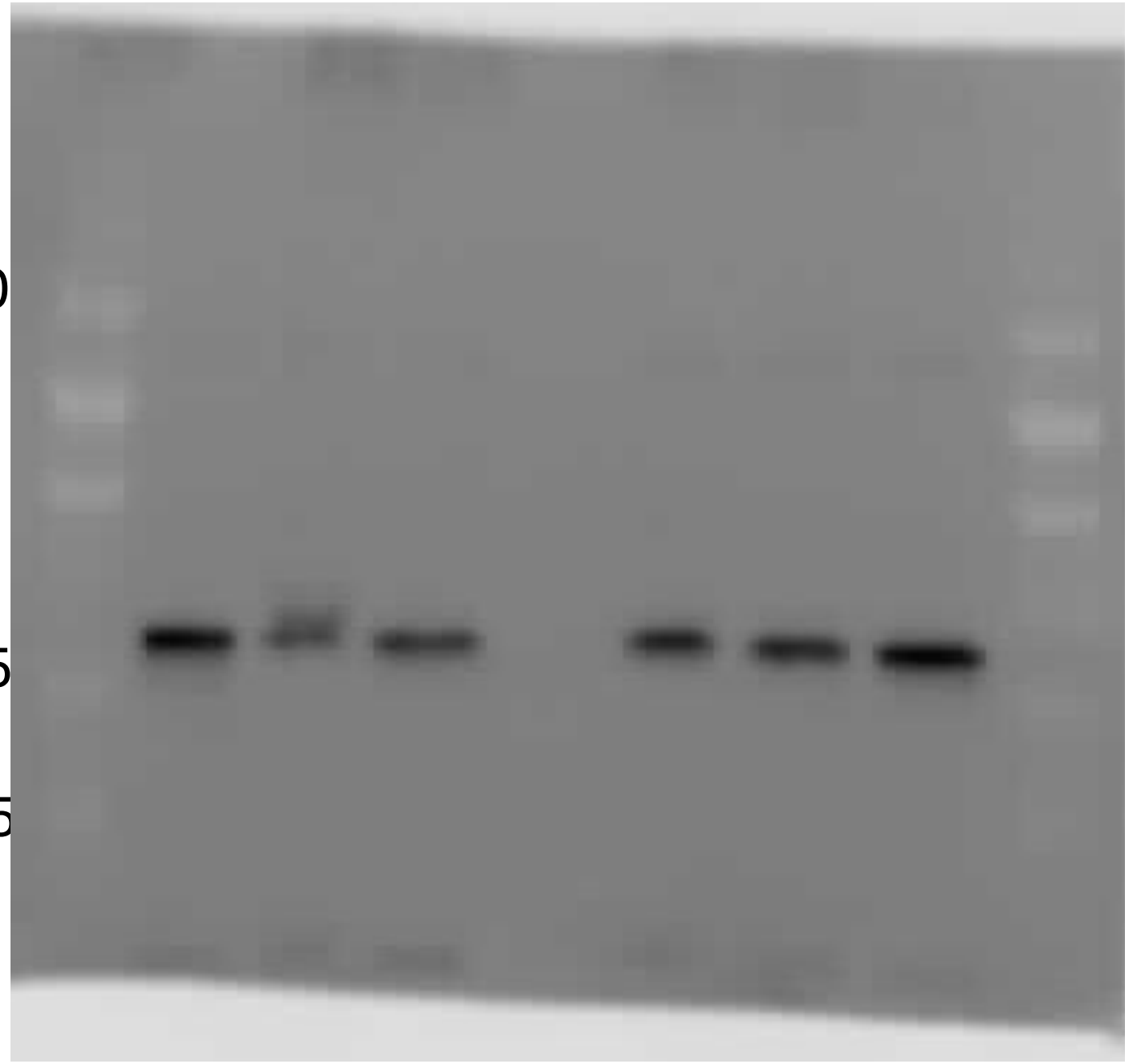

GAPDH @35kDa

FG0 BCL2 (Fig 3E)

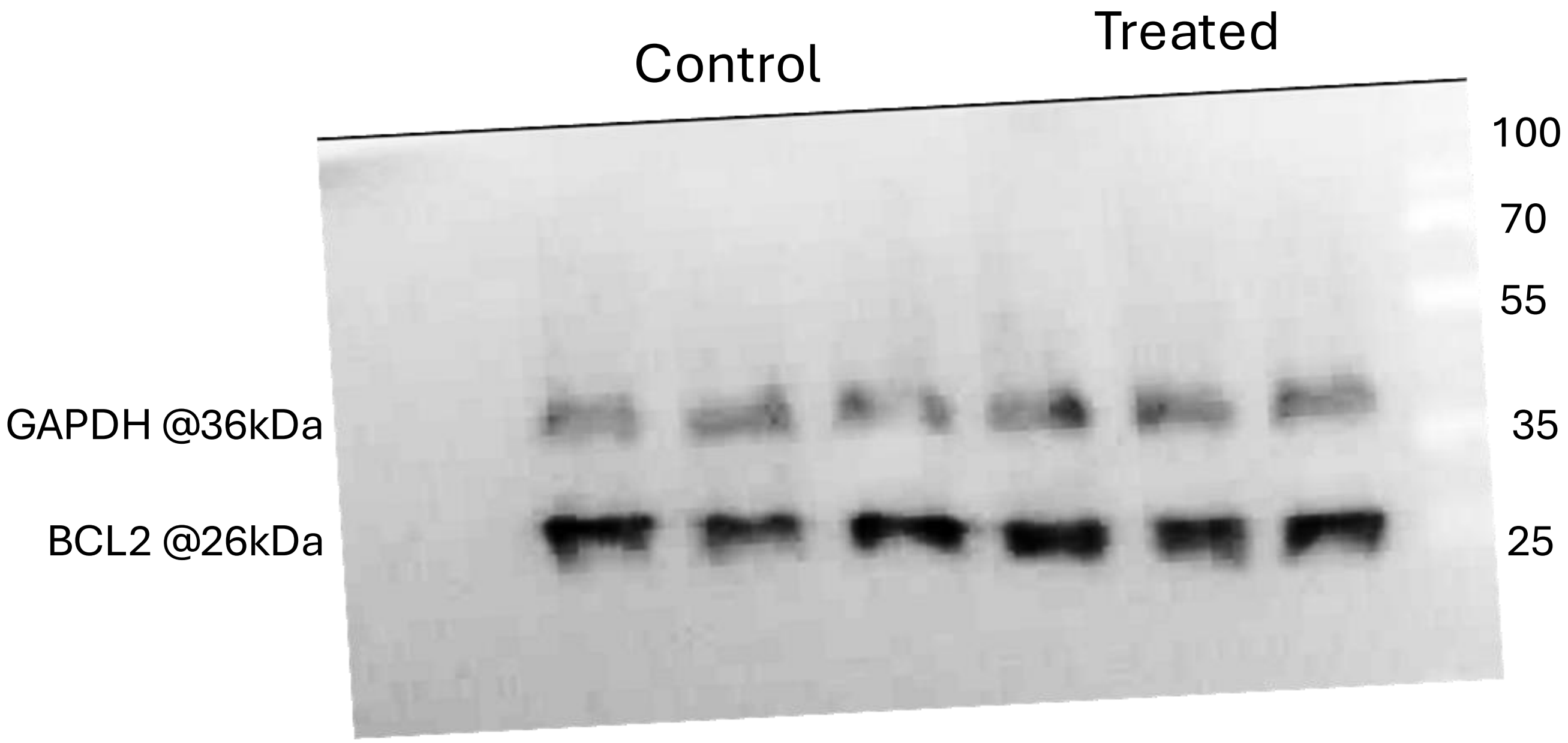

FG0 P53 (Fig 3F)

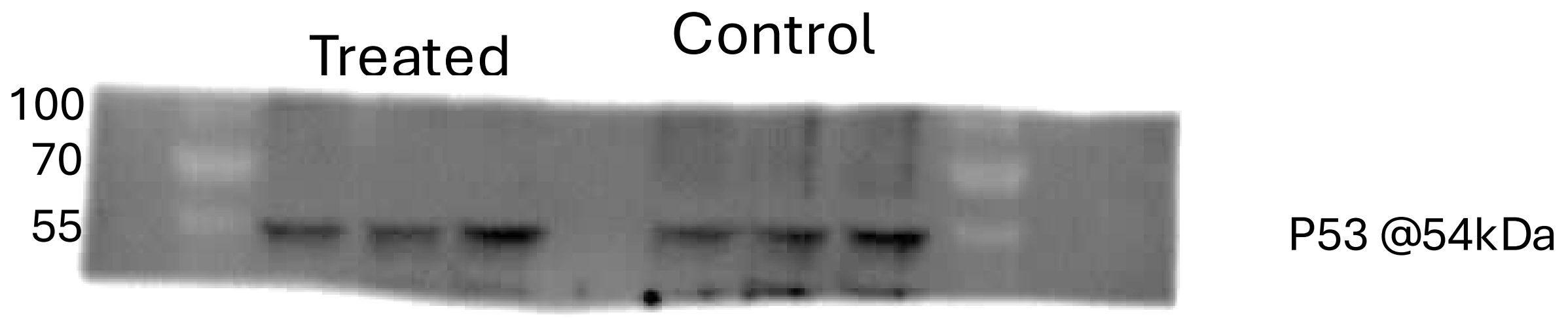

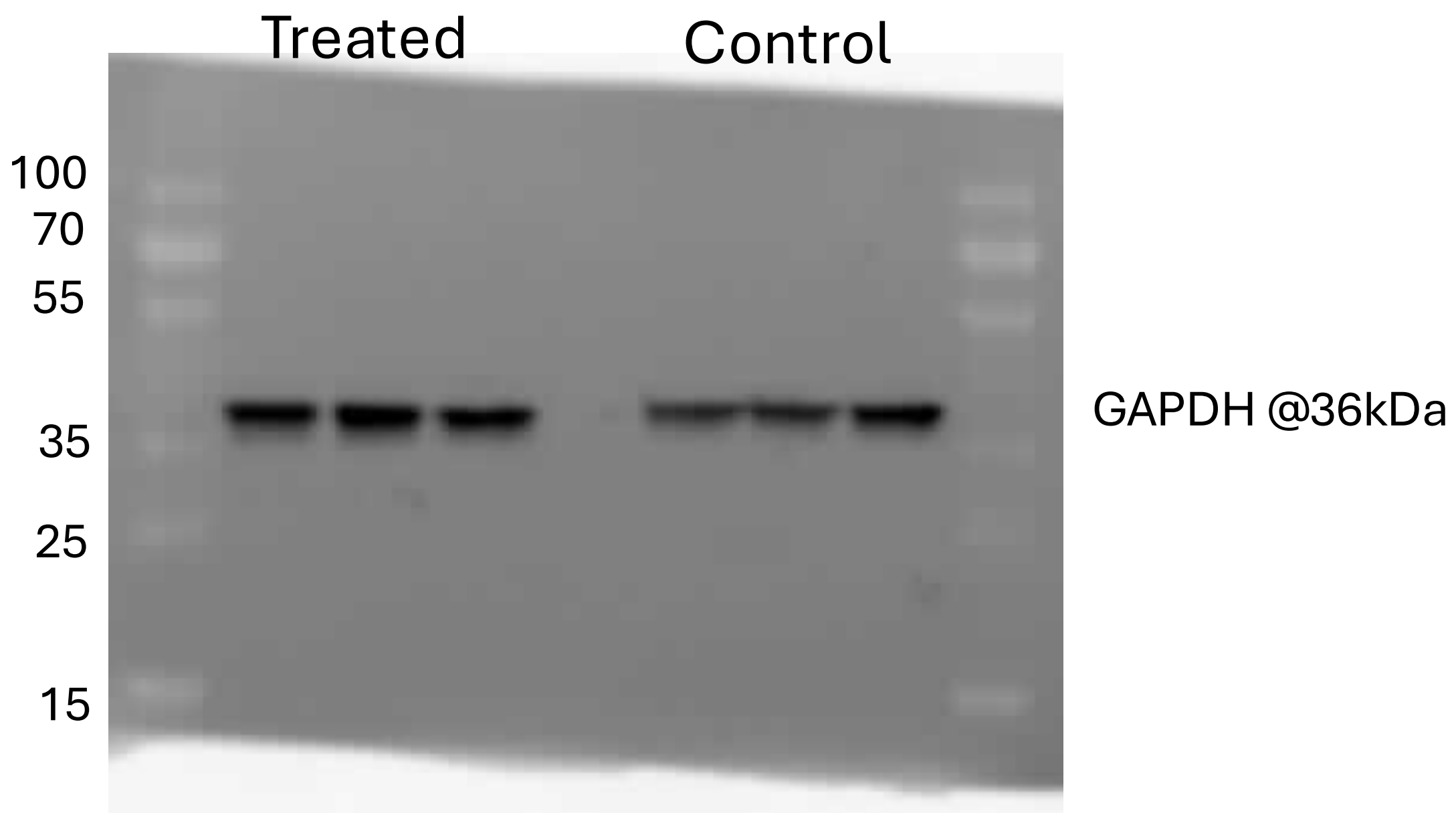

HT1080 Cyclin B (Fig 3 H)

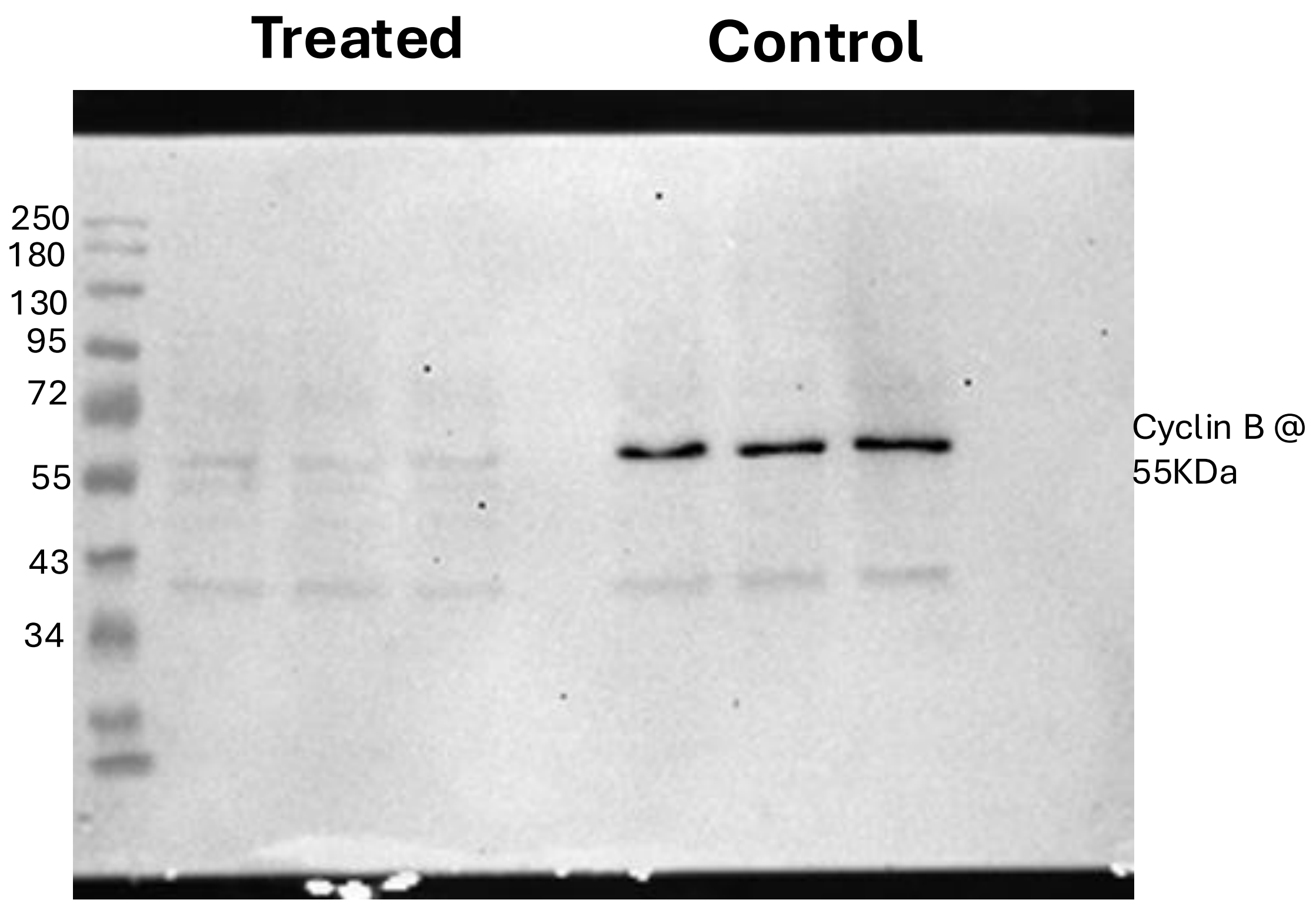

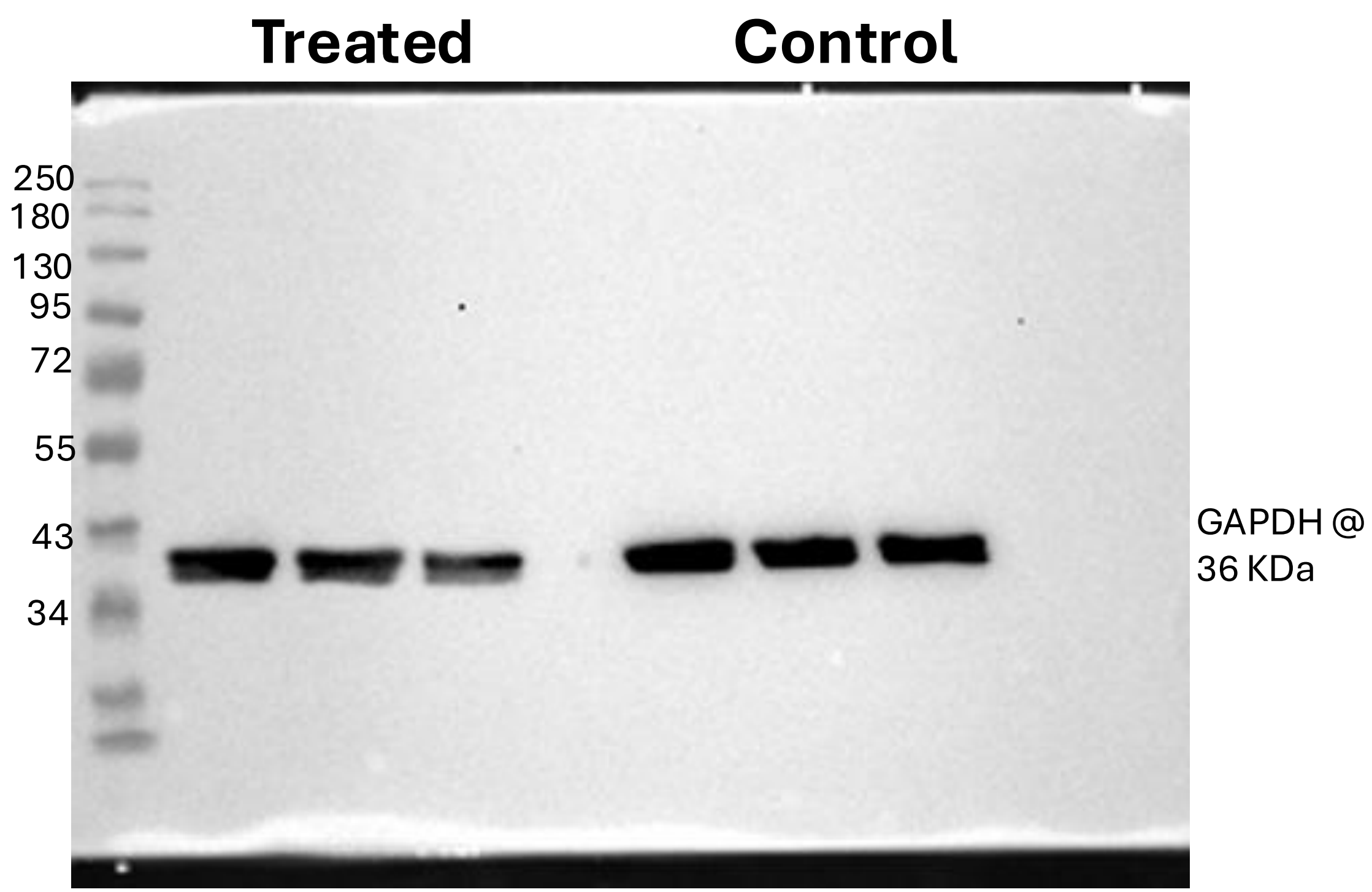

HT1080 BAG3 (Fig 3 I)

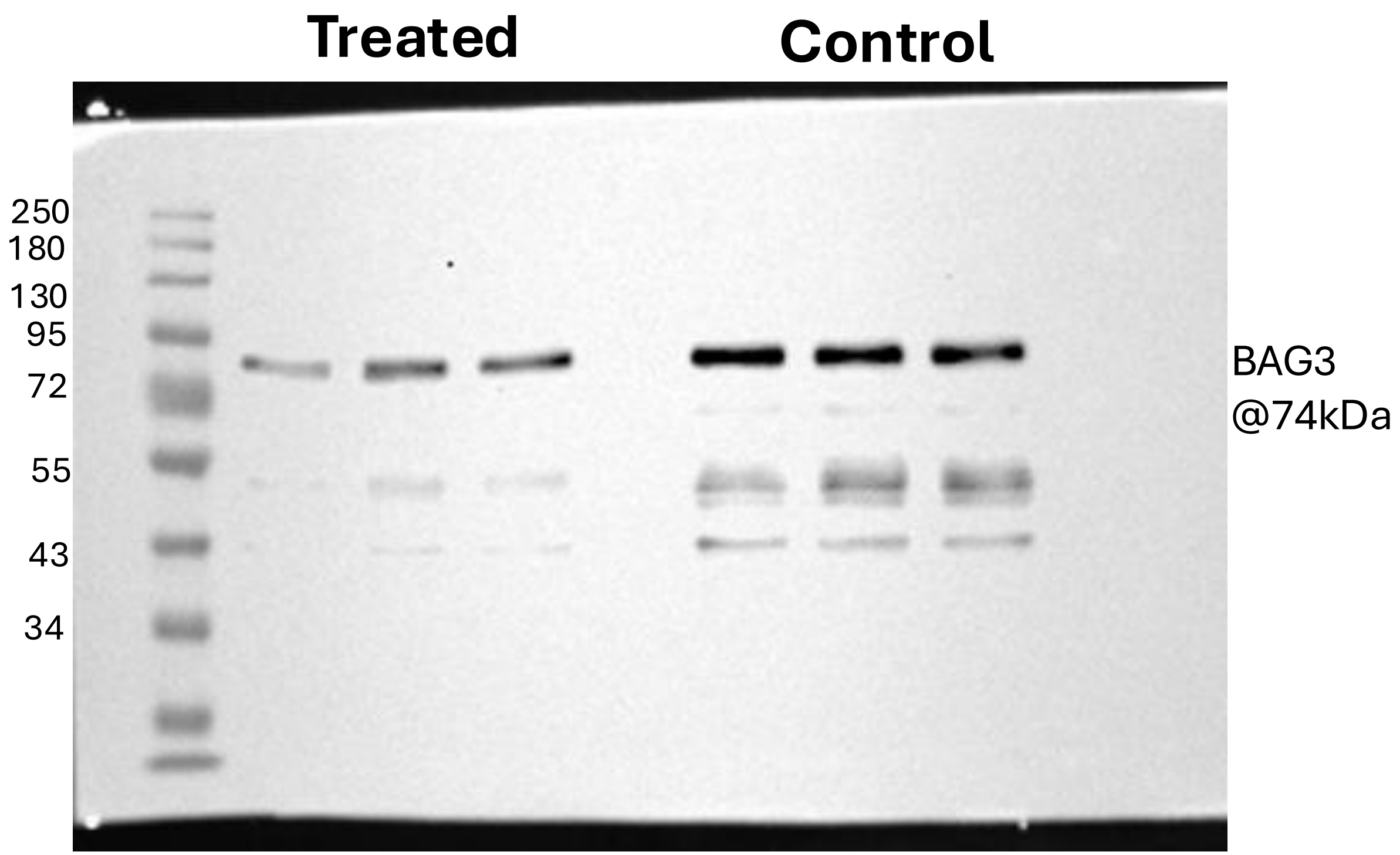

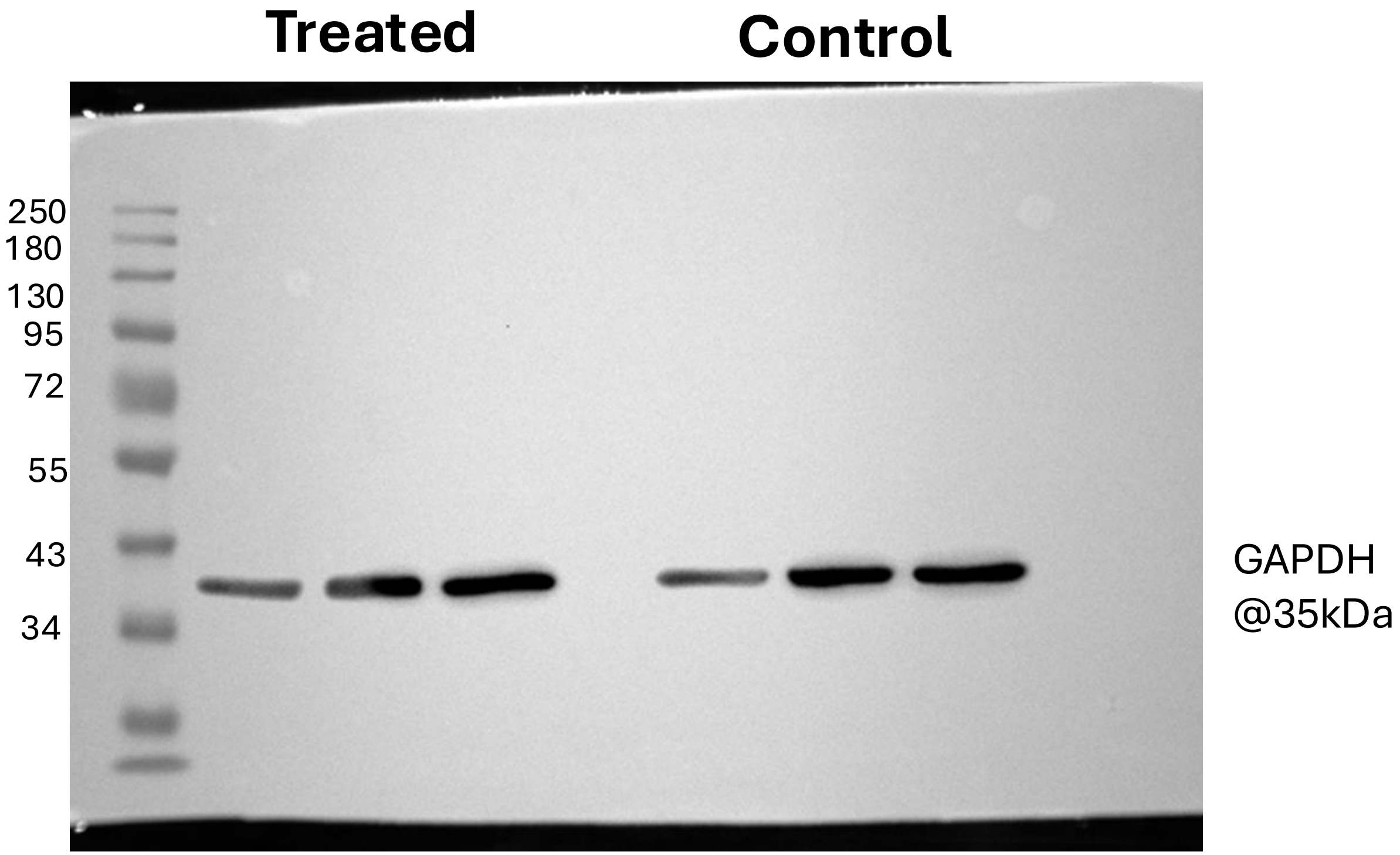

HT1080 P21 (Fig 3 J)

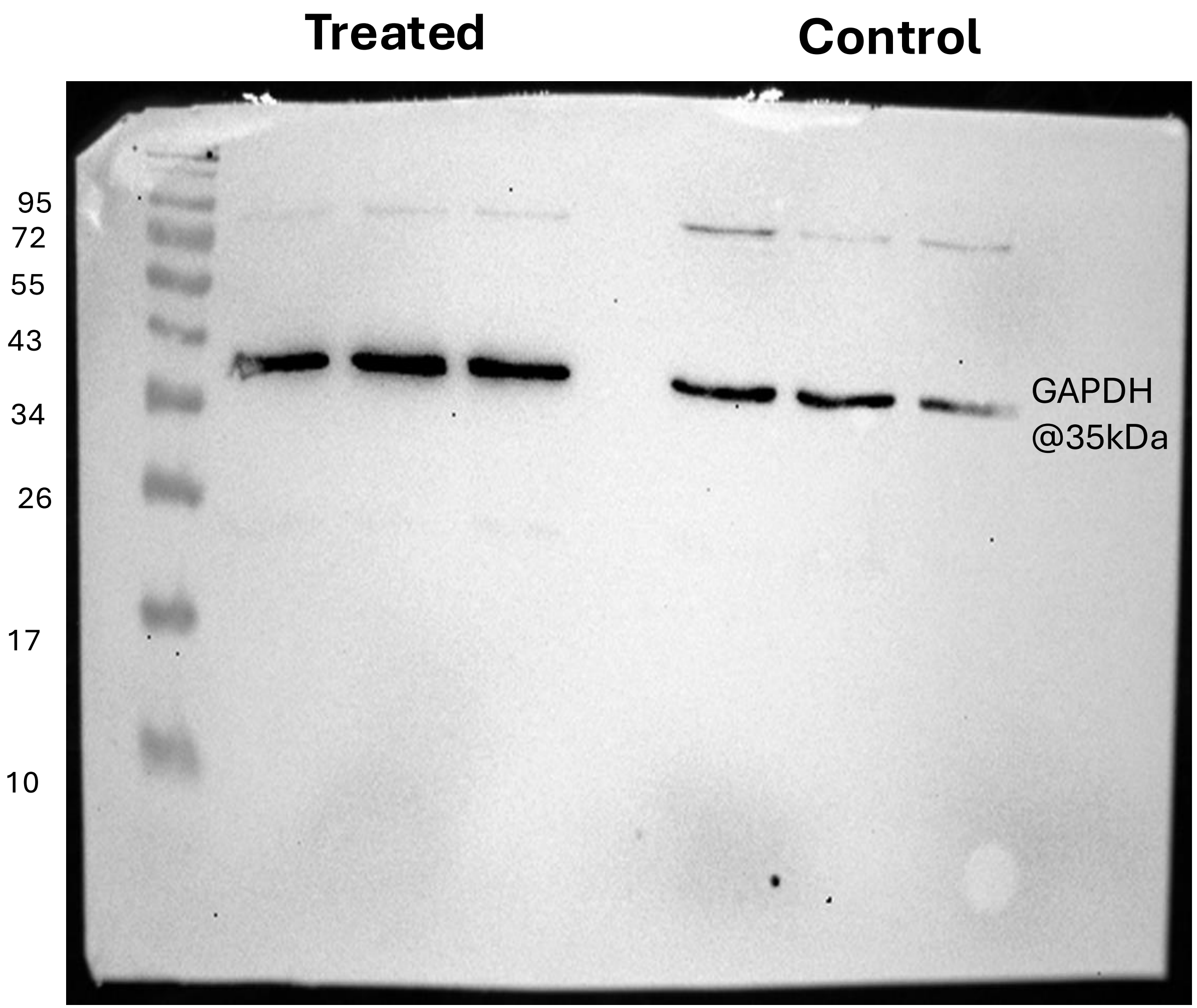

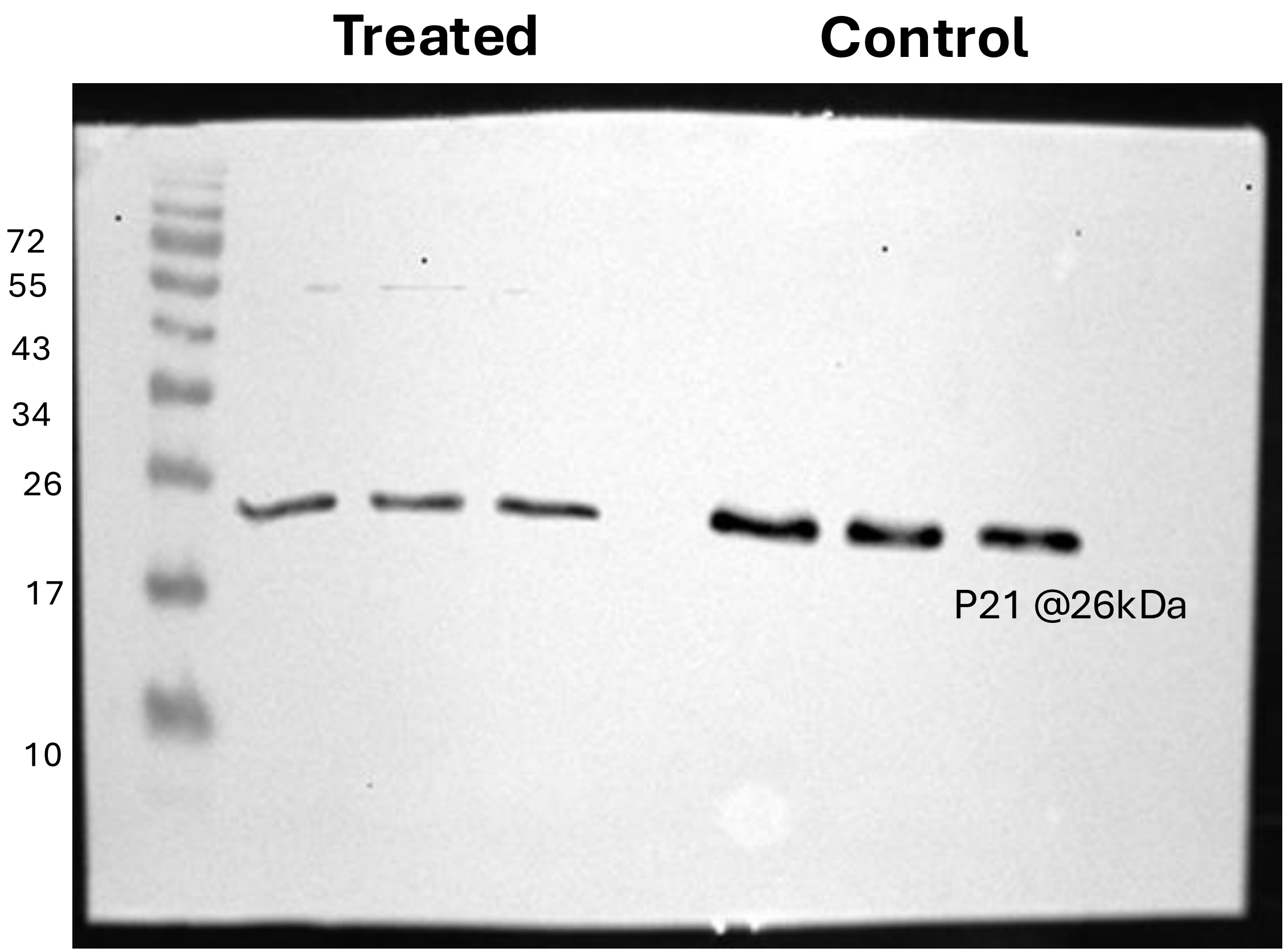

HT1080 BCL2 (Fig 3 K)

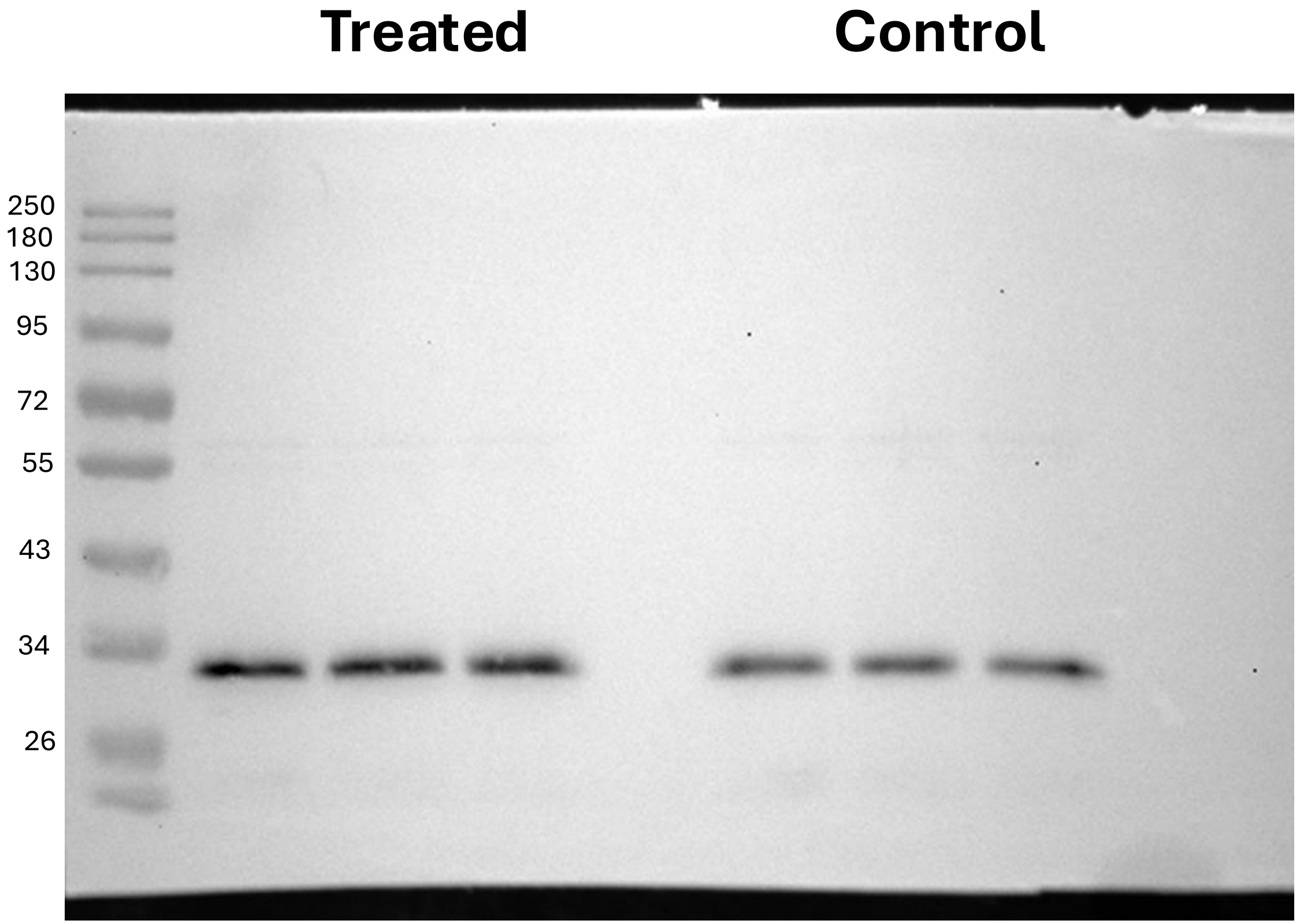

BCL2 @26kDa

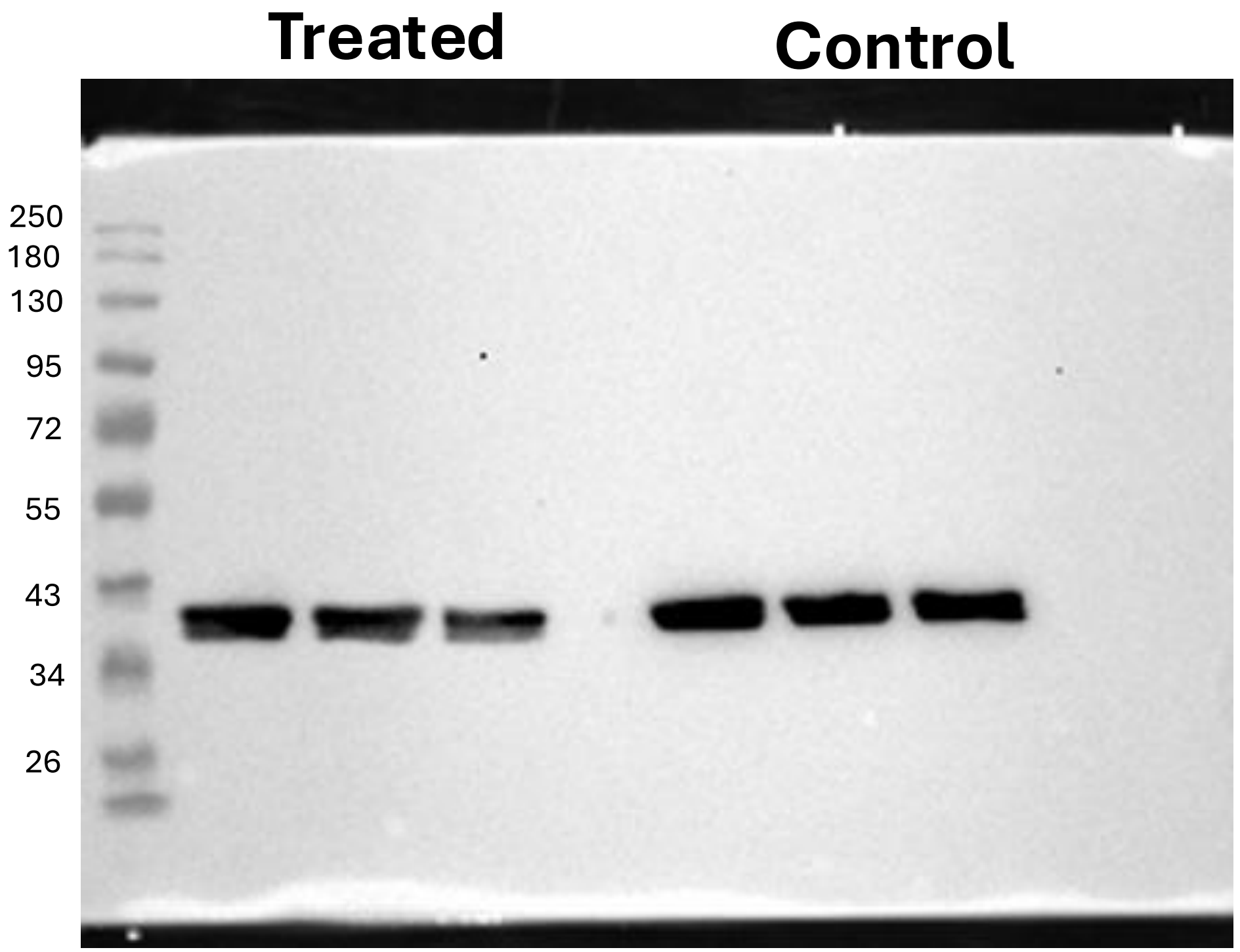

GAPDH @35kDa

HT1080 P53 (Fig 3 L)

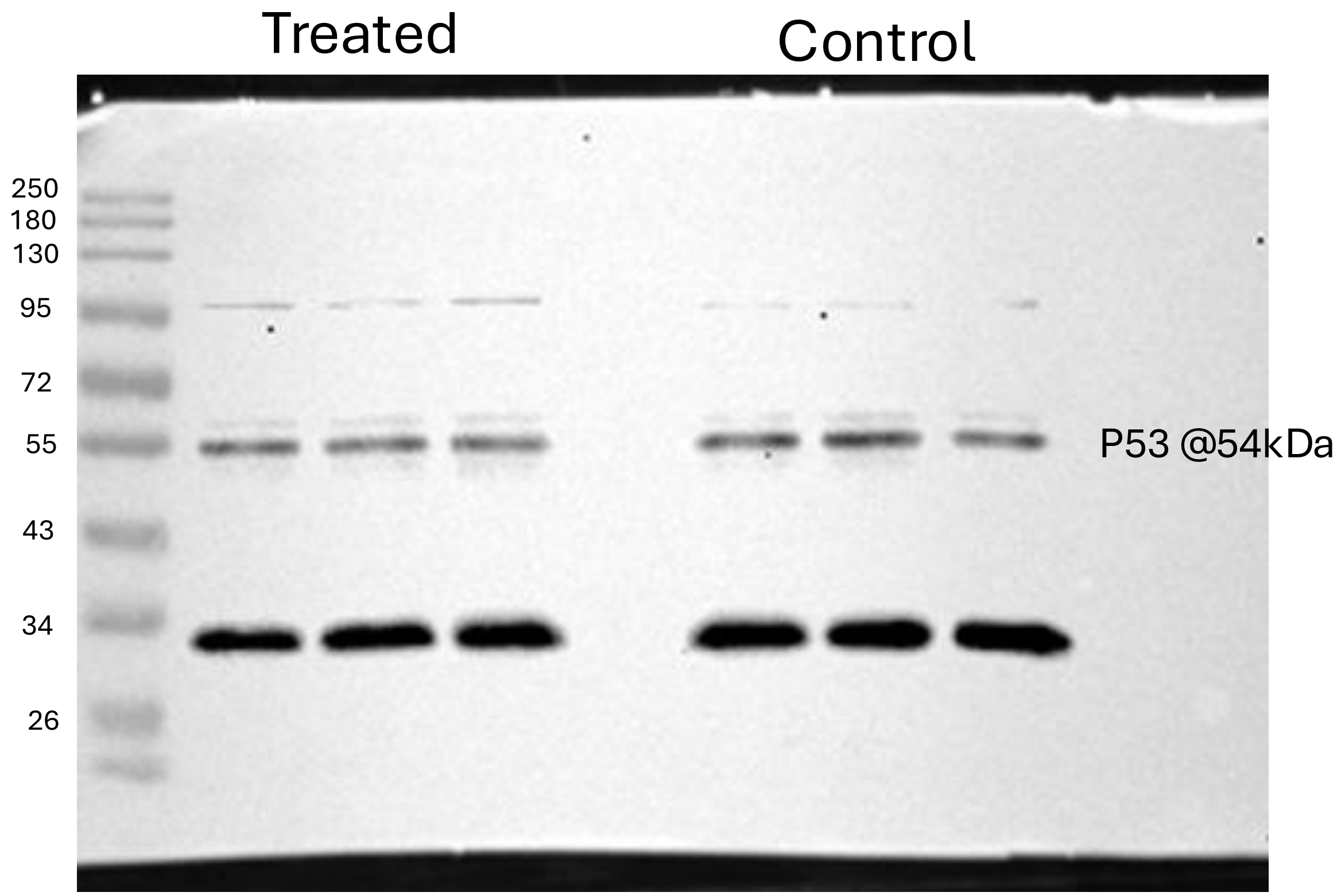

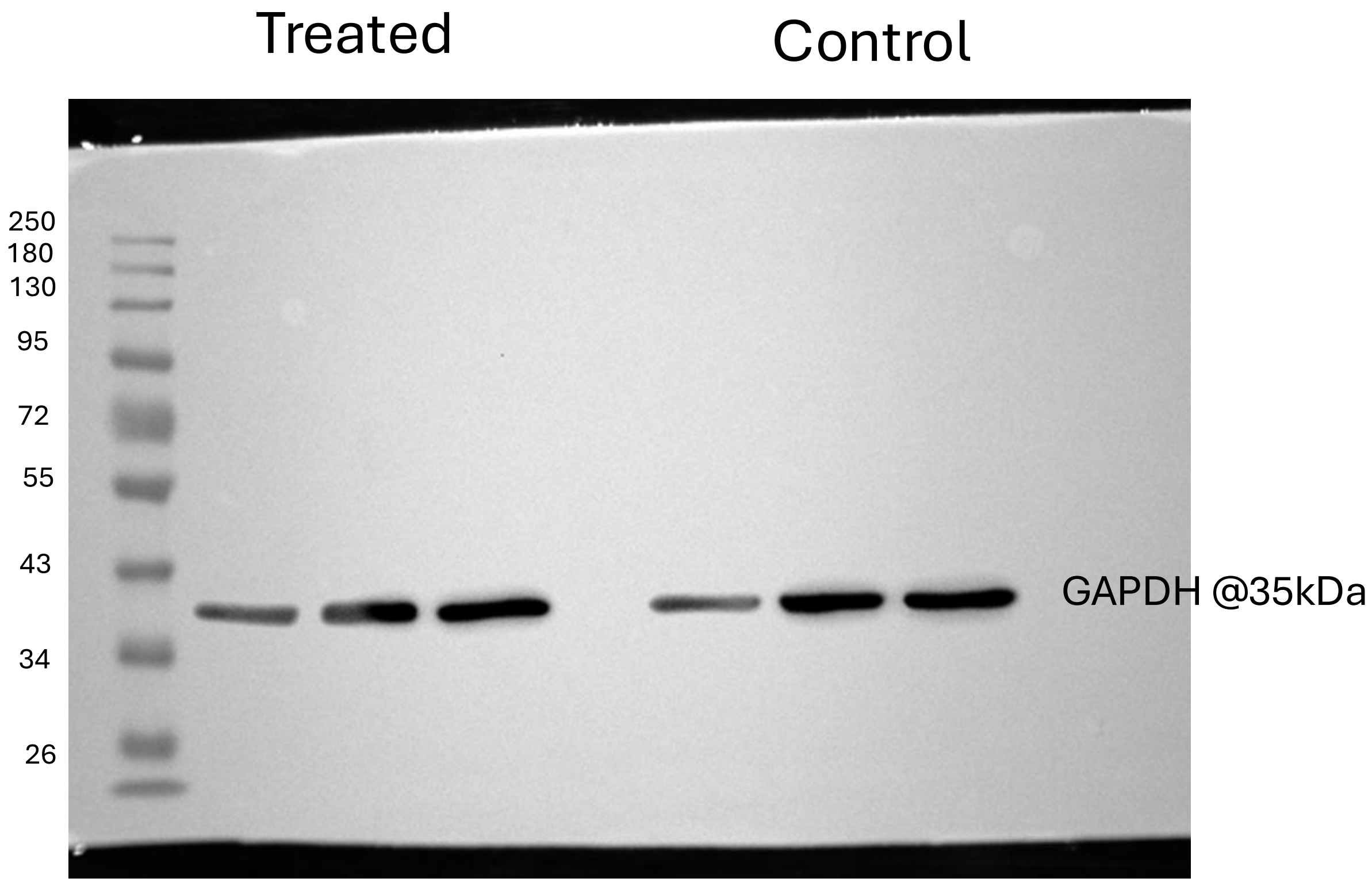

Supplement: Supplementary file 1 [file 42238_2026_481_MOESM1_ESM.pdf]
