# Supplementary material for: Cannabidiol selectively downregulates FAM111B expression and induces apoptosis in fibrosarcoma cells: an in vitro study
Source: J Cannabis Res. 2026 Aug 1;8:116. doi: 10.1186/s42238-026-00481-1 (PMC13721555; doi:10.1186/s42238-026-00481-1)
Supplement: Supplementary file 2 [file 42238_2026_481_MOESM2_ESM.docx]

**Supplementary information**

**Cannabidiol selectively downregulates FAM111B expression and induces apoptosis in fibrosarcoma cells: An in vitro study**

This supplementary information includes representative bright-field images of untreated and CBD-treated cells (Figure S1) and an independent manual-gating analysis of cell-cycle distribution (Figure S2), which complement the findings presented in the main manuscript.

**Figure S1: Representative bright-field images of untreated and CBD-treated FG0 and HT1080 cells following 24 h of CBD treatment.** Representative bright-field images (20× magnification) of untreated (Control) and CBD-treated FG0 fibroblasts (A) and HT1080 fibrosarcoma cells (B). Images are representative of three independent biological experiments.

**Figure S2: Independent manual-gating analysis of cell-cycle distribution following CBD treatment.**

Representative DNA-content histograms generated from manual gating of propidium iodide (PI)-stained FG0 cells (A) and HT1080 cells (C), with the corresponding quantitative analyses shown in (B) and (D), respectively. Cell-cycle distributions were analyzed independently by manual gating as a complementary approach to the Dean–Jett–Fox modeling algorithm used for the primary analysis presented in the main manuscript. Quantitative data are presented as mean ± SD from three independent biological experiments. Statistical analysis was performed using two-way ANOVA, followed by Šídák's post hoc multiple comparisons test. Manual gating showed trends comparable to those from the DJF model.
